# Organic-inorganic hybrid piezotronic bipolar junction transistor for pressure sensing

**DOI:** 10.1038/s41378-024-00699-0

**Published:** 2024-06-20

**Authors:** Emad Iranmanesh, Zihao Liang, Weiwei Li, Congwei Liao, Shunyu Jin, Chuan Liu, Kai Wang, Shengdong Zhang, Charalampos Doumanidis, Gehan A. J. Amaratunga, Hang Zhou

**Affiliations:** 1 Guangdong Provincial Key Laboratory of In-Memory Computing Chips, School of Electronic and Computer Engineering, Peking University Shenzhen Graduate School, Shenzhen, 518055 P. R. China; 2 School of Mechanical Engineering, Guangdong Technion-Israel Institute of Technology, Shantou, 515063 P. R. China; 3 State Key Laboratory of Microelectronics Device and Integrated Technology, Institute of Microelectronics, Chinese Academy of Sciences, Beijing, 100029 P. R. China; 4 Hefei National Research Center for Physical Sciences at the Microscale, University of Science and Technology of China, Hefei, 23000 PR China; 5 School of Electronics and Information Technology, Sun Yat-sen University, No. 132 East Waihuan Road, Guangzhou, 510006 P. R. China; 6 Department of Mechanical, Aerospace, and Biomedical Engineering, University of South Alabama, Shelby Hall, 3128, Mobile, AL 36688 USA; 7 Electrical Engineering Division, Department of Engineering, University of Cambridge, 9 JJ Thomson Avenue, Cambridge, CB3 0FA United Kingdom; 8 Zhejiang University, International Campus, Haining, China

**Keywords:** Electrical and electronic engineering, Sensors, Electronic devices

## Abstract

With the rapid development of the Internet of Things (IoTs), wearable sensors are playing an increasingly important role in daily monitoring of personal health and wellness. The signal-to-noise-ratio has become the most critical performance factor to consider. To enhance it, on the one hand, good sensing materials/devices have been employed; on the other hand, signal amplification and noise reduction circuits have been used. However, most of these devices and circuits work in an active sampling mode, requiring frequent data acquisition and hence, entailing high-power consumption. In this scenario, a flexible and wearable event-triggered sensor with embedded signal amplification without an external power supply is of great interest. Here, we report a flexible two-terminal piezotronic n-p-n bipolar junction transistor (PBJT) that acts as an autonomous and highly sensitive, current- and/or voltage-mediated pressure sensor. The PBJT is formed by two back-to-back piezotronic diodes which are defined as emitter-base and collector-base diodes. Upon force exertion on the emitter side, as a result of the piezoelectric effect, the emitter-base diode is forward biased while the collector-base diode is reverse biased. Due to the inherent BJT amplification effect, the PBJT achieves record-high sensitivities of 139.7 kPa^-1^ (current-based) and 88.66 kPa^-1^ (voltage-based) in sensing mode. The PBJT also has a fast response time of <110 ms under exertion of dynamic stimuli ranging from a flying butterfly to a gentle finger touch. Therefore, the PBJT advances the state of the art not only in terms of sensitivity but also in regard to being self-driven and autonomous, making it promising for pressure sensing and other IoT applications.

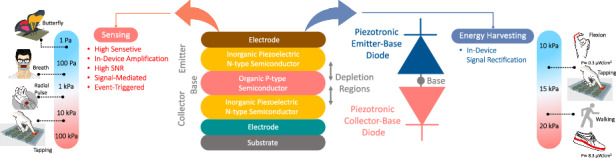

## Introduction

Wearable electronics, ranging from human-machine interaction devices to skin-like sensors, energy harvesting systems and health monitoring devices, are being rapidly developed^1–3^. Many advanced sensors have been reported and utilized in these applications such as to monitor human vital signs and generate power from human motions. Advances in sensors have also significantly progressed since design structure derivations can enhance the sensing performance in the detection of various parameters including pressure, strain, temperature, and humidity^4–9^. In these sensing applications, several techniques for obtaining high-gauge-factor devices have been reported^10–12^. One approach to realize an acceptable and accurate sensing mechanism is to obtain high signal-to-noise ratio (SNR) through the utilization of complex amplification and noise-reduction peripheral circuits although integration of an energy source and the associated power management remain challenging. An ideal route to address this issue is to construct a synergy between sensing and energy harvesting in which an event-triggered sensor is used to avoid frequent sampling and reduce power consumption and an energy harvesting mechanism is in place for energy recycling. In such a configuration, the signal from the sensing unit is the same as that captured by the energy harvesting system^13,14^. To realize such compact hybrid systems, several key challenges need to be overcome: first, rectification of the output current/voltage extracted from the signal originating from the sensing/harvesting device; second, amplification of the signal magnitude to make it suitable for use in wearable IoT nodes.

**Fig. 1:**
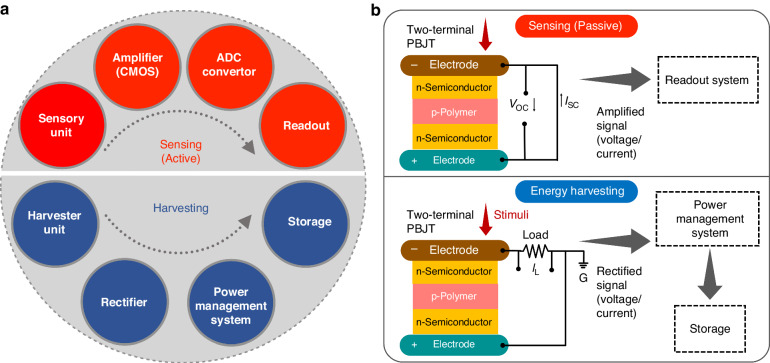
System-level approach to sensing and energy harvesting. **a** Schematic of piezoelectric sensing and harvesting systems along with required components. **b** Priority for the PBJT from the sensing unit to the harvesting system

A tradeoff between energy harvesting and sensing exists in that the output signal extracted for sensing in the form of a voltage or a current, needs to be amplified, whereas the harvesting function requires a reasonable magnitude of power (current $\times$ voltage) to drive the system. Figure 1a schematically illustrates a system-level approach toward typical piezoelectric-based harvesting/sensing units and shows the basic components of the aforementioned systems. Some techniques to integrate a diode-like rectifier into the sensors/nanogenerator units have been proposed^15^. In some piezotronic-based nanodevices, a pressure-induced piezoelectric potential can be used to modulate the current transport. This feature can be used to form an organic-inorganic junction as in a p-n structure to improve the sensing response. Having such a compact device with a fast response and low power consumption, where the extracted signal in either form (current/voltage) is rectified and amplified, is highly attractive. Piezotronic-based metal oxide semiconductor field-effect transistors (MOSFETs), where the gate is comprised of a piezoelectric material and used to modulate the channel current under pressure have also been demonstrated to fulfill this function^16–19^.

In terms of material selection, piezoelectric materials such as lead zirconate titanate (PZT), polyvinylidene difluoride (PVDF), and barium titanate (BTO), which have exhibited correlations between mechanical deformation and electrical polarization are of interest for hybrid harvesting/sensing systems. However, they also have several disadvantages of low output currents due to a high internal impedance and low polarization voltages corresponding to a high permittivity. Semiconductors coupled with piezoelectricity (piezotronics)^20^ have therefore been introduced to overcome the limitations of the low-current output in piezoelectric-based devices. Semiconducting piezoelectric materials, such as zinc oxide (ZnO), generate a higher output current density than that of insulating piezoelectric materials even though they suffer from a low piezoelectric potential due to screening effects^21^. In this scenario, piezotronics theory has been employed to demonstrate some promising devices, including AC/DC nanogenerators and wearable sensors^22,23^. The piezoelectric performance of several semiconductors, such as ZnO, gallium nitride (GaN), indium nitride (InN), cadmium sulfide (CdS) and monolayer MoS_2_ have been reported^24–28^.

Most piezoelectric sensing systems require extra complex circuitry for amplification. Complementary metal-oxide semiconductor (CMOS) technology and its recent advances, in this case, are mature enough to be utilized in amplification. However, interfacing and flexibility of CMOS-based devices are still under development. Therefore, a compact device without any extra interfacing or amplification circuits is in great demand. Above all, for wearable devices, most piezotronic-based sensing devices previously reported have been neither autonomous nor highly sensitive. Bipolar junction transistors have been shown to be able to meet some of the operational requirements of a self-driven and self-powered sensor^29^.

In this work, a novel device combining pressure sensing with energy harvesting based on a piezotronic n-p-n bipolar junction transistor (PBJT) is proposed and studied. It is a mechanically-driven, organic-inorganic hybrid, vertically-stacked n(collector)-p(base)-n(emitter) structure. The PBJT is a signal-mediated device (providing both a current and a voltage) and in-device signal rectification/amplification is embedded in an integrated architecture. Figure 1b presents the priorities for the PBJT in sensing and harvesting applications along with components required to construct the system. From a sensing perspective, (the focus of this paper), the proposed PBJT, as a passive device, has the champion sensitivity. It is capable of detecting ultralow dynamic stimuli over a broad range from a flying butterfly to a gentle touch, while functioning in three distinct regimes (off-state, saturation, and on-state). From an energy scavenging standpoint, the PBJT generates an output peak power density of 8.3 µW/cm^2^ as a wearable energy harvester (the PBJT is attached to a shoe insole and this power is generated during 100 cycles of gentle walking), which seems promising for powering some low-power electronic devices^30^. The following sections of this paper are classified as follows: device fabrication and material characterization, theory of operation and working principles, and device characterization with dynamic/static response, The PBJT as an event triggered sensing unit, the PBJT pixelated array and its applications will be discussed in detail.

## Results

### Device fabrication and material characterization

**Fig. 2:**
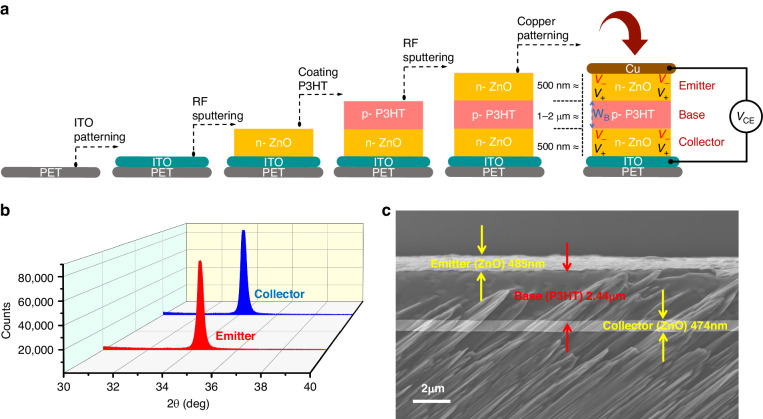
Device fabrication and characterization. **a** Schematic of the fabrication steps of the n-p-n structure in which a *p*-type polymer is sandwiched by two n-type piezoelectric semiconductor materials, with thickness differentiation between the layers. **b** XRD image of PBJT showing the orientation of wurtzite crystals of ZnO. The two piezoelectric semiconductor layers of ZnO at the top and bottom are labeled as the emitter, and the collector respectively. **c** SEM cross-sectional view of the fabricated device

The fabrication steps for obtaining a vertically-stacked structure of an n-p-n transistor in which a p-type polymer, poly (3-hexylthiophene-2,5-diyl) (P3HT), is sandwiched between two piezotronic *n*-type ZnO layers are schematically given in Fig. 2a. The first step starts with an ITO electrode being prepared by DC sputtering to form the bottom electrode on a PET substrate. Then, ITO/PET is used as a substrate to deposit the first ZnO layer. The ZnO bulk is RF sputtered while the pressure is maintained at 0.7 ~ 0.77 Pa (the thickness of ZnO is 420–500 nm). Afterwards, annealing of the thick ZnO layer forms the emitter. Next, P3HT is coated as a uniform p-type polymer with a thickness of 1–2 μm on top of the PET/ITO/ZnO substrate via a doctor blade process. Several p-type semiconductor polymers, such as: P3HT, poly (3,4-ethylenedioxythiophene)-poly (styrene sulfonate) (PEDOT:PSS), and poly (triarylamine) (PTAA), can be utilized to form the base. In the fabrication process, when using PEDOT:PSS, the ZnO layer is etched at the collector side due to the acidity of PEDOT:PSS. Although dilution of PEDOT:PSS may overcome this issue, PEDOT:PSS is not an appropriate candidate for sensing applications. The material selected for the base can vary depending on the applications such as sensing (P3HT, higher open-circuit voltage) or harvesting (PEDOT:PSS, higher power density). Due to its biocompatibility, cost-effectiveness, availability, and ease of preparation, P3HT has been selected for fabrication. To form the top ZnO layer (collector), RF-sputtering with the same settings as mentioned earlier for the bottom-side ZnO is considered. To obtain the desired orientation of wurtzite crystallization of ZnO, the device is annealed. Finally, the top electrode (copper) is patterned through DC-sputtering. Comprehensive details of the fabrication processes (n-p-n and p-n junction) are given in the Materials and Methods section and Figure S1a. ZnO as a piezoelectric semiconductor is supposed to be a proper candidate for pressure/strain detection under low frequencies <100 kHz^31^. Analysis verified that the piezoelectricity of ZnO film is optimized if the crystals are well-oriented in the 002 direction. The hexagonal shape of ZnO bulk wurtzite crystals is illustrated in Figure S1b^32^. The crystal orientations of the ZnO films in the n-p-n transistor were studied by X-ray diffraction (XRD) (Fig. 2b). The significant peaks at 34.8° are evidence of the orientation of the ZnO films in the 002 direction.

The cross-sectional SEM image of the device (Fig. 2c) reveals the thickness and uniformity of each layer, verifying that a junction was firmly formed for the top (emitter) and bottom (collector) ZnO films with the P3HT base region. A piezotronic p-n junction diode obtained through the formation of an organic-inorganic junction was also studied and compared with the n-p-n bipolar junction transistor (Fig. S1).

### Theory of the PBJT and principle of operation

Bipolar junction transistors in the n-p-n or p-n-p form for analogue and digital electronic circuits are well established (amplifiers and switches). However, the piezotronics theory of BJT as an autonomous sensing unit capable of transient voltage and current generation is still in a very early stage. In this section, the operation of the piezotronic n-p-n BJT as a sensor is discussed.

The sensor has the structure of a bipolar junction transistor composed of ZnO and P3HT. ZnO is a semiconducting piezoelectric material and has the properties of an n-type semiconductor as deposited in its intrinsic form^33^. P3HT is a semiconducting conjugated polymer with p-type properties. The PBJT differs from the conventional BJT in its transient response in that “control” of the base occurs through an applied force and generated charges, sequentially. Unlike conventional BJTs, here, both the emitter and the collector are highly n-doped compared to the p-type base. Therefore, the depletion regions at the emitter-base and collector base-junctions extend mainly into the P3HT base. The base is floating, with the collector and the emitter connected through a measuring system or a load. The collector forms the bottom of the device contacted by ITO and is placed on a rigid base. The emitter contacts Cu at the top of the device, which forms the surface of the sensor.

**Fig. 3:**
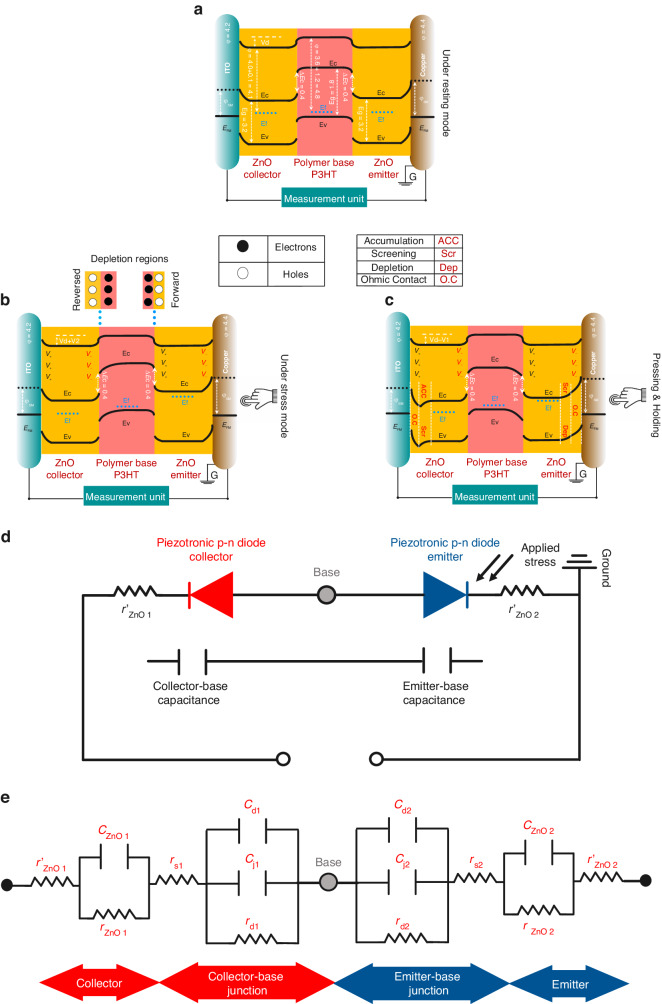
Energy band diagrams, and working mechanism of the piezotronic bipolar junction transistor along with its electrical and capacitance-resistance (c**–**r) equivalent circuits. **a** Schematic illustration of the working mechanism of the device and the conduction band level under no stress, (**b**) under applied stress, and (**c**) under applied stress and holding. Piezoelectric-induced electrons flow from Cu to ITO, where a new equilibrium state is created in which the piezoelectric potential is screened by free carriers in the ZnO film. **d** Electrical equivalent circuit diagram of the n-p-n structure including two pieztronic diodes back to back. **e** Small-signal equivalent circuit of the PBJT (c-r)

The device in resting mode (in the absence of any applied pressure) is illustrated in Fig. 3a. When a force stimulus is applied to the ZnO emitter, it is transferred through the device to the ZnO collector as well. Uppon application of a mechanical load, the ZnO becomes polarized through the piezoelectric effect such that the emitter at the contact is negatively-charged (-) and the collector at the contact is positively-charged (+). This in essence translates into the application of a collector- emitter voltage +${V}_{{CE}}$ to the transistor.

As in a conventional bipolar transistor, the piezoelectrically-generated ${V}_{{CE}}$ dropped across the reversely biased collector (ZnO)-base (P3HT) n-p junction and the forward-biased base-emitter p-n junction ${(V}_{{CE}}={V}_{{CB}}+{V}_{{BE}})$. Figure 3b shows the band diagram immediately after the application of a mechanical load. There is a transient current, ${I}_{{CE}}(t)$, which results from the ‘turn-on’ of the symmetric base-emitter, and collector-base heterojunctions, and has a displacement current component, ${I}_{{CEDis}}\left(t\right)={C}_{C-B}\left(t\right).\frac{d{V}_{{CB}}(t)}{{dt}}+{V}_{{CB}}\left(t\right).\frac{d{C}_{C-B}(t)}{{dt}}={C}_{B-E}\left(t\right).\frac{d{V}_{{BE}}(t)}{{dt}}+{V}_{{BE}}\left(t\right).\frac{d{C}_{B-E}(t)}{{dt}}$, where ${C}_{C-B}(t)$ and ${C}_{B-E}(t)$ are the collector-base and base-emitter depletion region capacitances, respectively. They are time dependence during the transient ${V}_{{CE}}(t)$ and associated with an expanding depletion region at the collector-base junction (reverse biased) and a contracting depletion region at the base-emitter junction (tending to being forward biased). The continuity of ${I}_{{CEDis}}\left(t\right)$ through the device (floating base) requires the transient “turn-on” of the base emitter junction to allow the current to flow through the emitter. The transient turn-on of the base-emitter junction leads to electron injection into the base from the heavily doped ZnO emitter. These injected electrons can be effectively swept out of the base into the collector due to the collector-base depletion capacitance which extends into the base. This transient bipolar action results in a component of conduction current component, ${I}_{{CECond}}\left(t\right)$, in addition to that due to the displacement current component, ${I}_{{CE\; Dis}}\left(t\right)$, associated with the formation of the collector–base and base-emitter depletion regions.

Assuming that ${V}_{d}$ is the potential difference between the two symmetrical heterojunction structures due to the formation of piezotronic n-p and p-n diodes, ${V}_{d1}$, ${V}_{d2}$ are assumed to be the piezoelectric generated voltages due to force applied on the collector and emitter sides respectively. If the pressure is sufficiently high and the developed ${V}_{{BE}}$ is greater than the “turn-on voltage” of the emitter junction, i.e., ${V}_{{BE}} > {V}_{{BE\; ON}}$ (0.3–0.4 V from Fig. 3a–c), then at some point in the transient, “turning-on” of the base-emitter junction can occur and electrons are injected into the base. Hence, the initial output current is amplified, as in a BJT.

As explained earlier, this transient bipolar action results in a conduction current component, ${I}_{{CECond}}(t)$, in addition to the displacement current. Therefore, a higher transient current, ${I}_{{CE}}\left(t\right)={I}_{{CE\; Dis}}\left(t\right)+{I}_{{CE\; Cond}}(t)$ is achieved in the ZnO bipolar transistor piezoelectric sensor resulting in a higher sensitivity (current-based) compared to other structures. With time, the carriers (electrons) in the ZnO collector and emitter will adjust to screen the polarized charge, giving rise to ${V}_{{CE}}(t)$, and the device will return to the equilibrium state with excess electrons at the bottom contact to screen the positive polarized charge, and with a depletion region at the top contact to screen the negative polarised charge (Fig. 3c). As is well known in BJT physics, the n-p-n BJT acts as two n-p diodes placed back-to-back in series. A contrast of the mechanism of a p-n junction diode and an n-p-n transistor is given in the Supplementary Information (Fig. S2). A detailed study of the p-n junction mechanism and energy band diagram are given in Fig. S2a, b. The equivalent circuit of the piezotronic p-n junction diode is also illustrated in Fig. S2c.

Figure 3d shows a schematic of the n-p-n circuit diagram from an electrical standpoint. According to a circuit level approach, upon exertion of stress on the emitter side, the two aforementioned back-to-back diodes are differently modulated with respect to the polarization direction. Therefore, the collector-base diode is reverse biased (red) and the emitter-base diode is forward biased (blue). The contact resistances of the system due to the electrodes and wiring are given as $${{r}^{{\prime} }}_{{ZnO}1}$$, and $${{r}^{{\prime} }}_{{ZnO}2}$$. Two capacitances in series related to the collector-base and emitter-base diodes are also shown. Figure 3e depicts a typical small-signal capacitor-resistor (c-r) circuit of the PBJT. The total current at the p-n junction is the summation of the electron and hole currents that remain steady through the depletion area. The gradients from the minority carrier concentrations create diffusion currents, and since the electrical field is assumed to be zero at the space-charge edge, the drift current for the minority carrier in this approach can be ignored. From the c-r circuit stand point, the aforementioned two p-n diodes can be explained as follows: junction capacitances, ($${c}_{j1}$$,$$\,{c}_{j2}$$), are placed in parallel with the diffusion resistances ($${r}_{d1},{r}_{d2})$$) and diffusion capacitances ($${c}_{{d}_{1}}$$,$${c}_{{d}_{2}}$$). Two capacitors and two resistors in parallel related to the ZnO bulks on the emitter and the collector sides ($${c}_{{ZnO}1}$$, $${r}_{{ZnO}1}$$_,_ $${c}_{{ZnO}2}$$, $${r}_{{ZnO}2}$$) are added in series to the circuit. The neutral n and p regions in each diode have a numbers of resistances, so the actual p-n junction includes a series resistance that completes the equivalent circuit. Resistors of $${{r}_{s}}_{1}$$, and $${{r}_{s}}_{2}$$, corresponding to the contact resistance are also placed in series. The equivalent circuit of the n-p-n structure (Fig. 3e) presents a series of capacitances that consequently decreases the overall built-in capacitance. The collector-emitter electric potential, $${V}_{{CE}},$$ is modeled in terms of the generated piezoelectric charges and in-series capacitances of the collector-base and the emitter-base junctions (in an ideal case of a diode, the junction resistances are negligible). This model enhanced obtained voltage of the PBJT compared to that of piezotronic Schottky contacts (such as: ITO/ZnO/Cu), and piezotronic p-n junctions (such as: ITO/ZnO/P3HT/Cu) due to the introduction of in-series capacitances (capacitances introduced by the formation of junctions, contacts and materials are called built-in capacitances while the system capacitance refers to the variation in the overall built-in capacitance due to any applied stimuli in this paper). The reduction of the PBJT overall built-in capacitance compared to the aforementioned junction/Schottky-based devices under a constant applied pressure results in voltage amplification. Hence, the PBJT also offers record-high sensitivity with respect to the voltage.

1 $${V}_{CE}=\left(\frac{Q}{\left(\frac{{C}_{C-B}\cdot {C}_{E-B}}{{C}_{C-B}+{C}_{E-B}}\right)}\right),$$

The total piezoelectric generated charges of the collector and emitter, ($${Q}_{{Total}})$$) can be obtained as:

2 $${Q}_{Total}={d}_{33}\cdot {F}_{33}+\left(\frac{l}{t}\right){d}_{31}\cdot {F}_{31},$$

where *d*_*33*_ and *d*_*31*_ are the piezoelectric coefficients in the 33 and 31 directions respectively. ${F}_{33}$, and ${F}_{31}$ are the applied forces in the respective directions; and *l* and *t* are the length and thickness of the collector/emitter piezotronic layers.

The collector-emitter current, ${I}_{C}$ can also be modeled based on diffusion theory^34,35^, by referring to Fig. 2a as:

3 $${I}_{C}=\frac{{A}_{E}q{D}_{n}{n}_{PBO}}{{L}_{n}}\,{{Cos}}\,{ech}\left(\frac{{W}_{B}}{{L}_{n}}\right)\exp \left(\frac{q{V}_{BE}}{kT}\right),$$

where *A*_*E*_ is the cross-sectional area of the emitter-base junction, the absolute value of the unit electronic charge is given by *q*, *W*_*B*_ is the width of the base region, and *n*_*PB0*_ is the thermal equilibrium electron concentration in the *p*-type semiconductor. ${L}_{n}=\sqrt{{D}_{n}{\tau }_{n}}$ is the diffusion length of holes and *D*_*n*_ is the diffusion coefficient for electrons. Boltzmann factor is k and *T* is the temperature in Kelvin. ${V}_{{BE}}$ is the base-emitter voltage and the collector-emitter voltage is coupled to the base-emitter and base-collector (${V}_{{CE}}={V}_{{BE}}+{V}_{{CB}}$). The modeling and numerical analysis of the piezotronic n-p-n bipolar junction transistor are detailed in Supplementary Information S3.

Bipolar junction transistors as three-terminal devices can be operated in three different configurations: common base, common collector, and common emitter, based on which of the three main components of the bipolar transistors (base, collector, and emitter) are grounded. In the common emitter configuration, the emitter terminal is the common output and input channel. The base-emitter terminal is configured to be the input while the collector-emitter terminal is taken as the output. The common emitter configuration is mostly utilized in various applications due to the amplification of both the current and voltage. The working principles of the common emitter configuration is that the base-emitter region is biased by ${V}_{{BE}}$, while ${V}_{{CE}}$ is applied across the emitter-collector region. A collector current (output current) is then observed across the emitter-collector region and can be labeled as ${I}_{C}$. The output side is reverse biased, whereas the input side is forward biased. Since the base-emitter region acts like a forward-biased diode, its depletion region will be very narrow, whereas the collector-base reverse-biased diode has a wider depletion region. The input characteristics of the transistor can be interpreted as the corresponding relation between the input current and input voltage. The output characteristics of the device in this case are the relationship between the output current and output voltage while the input current is kept constant. Such a bipolar transistor traditionally functions statically in three regions: active, saturation and cut off regions. In the active region, as the output voltage increases, there is no noticeable change. In the cutoff state, the input current is less than zero, and once both junctions are forward-biased, the device works in the saturation mode. To satisfy the above theory, the emitter is grounded and the base-collector region is biased with a typical constant DC voltage of 0.7 V. The current in the base region labeled as ${I}_{B}$, turns on the device to shift to the active state from the cut off mode. Therefore, the in-flowing static current (base current), (${I}_{B}$) was modified to illustrate that the output characteristics of the transistor are independent of the bias voltage^36^.

### Device characterization and dynamic/static response

**Fig. 4:**
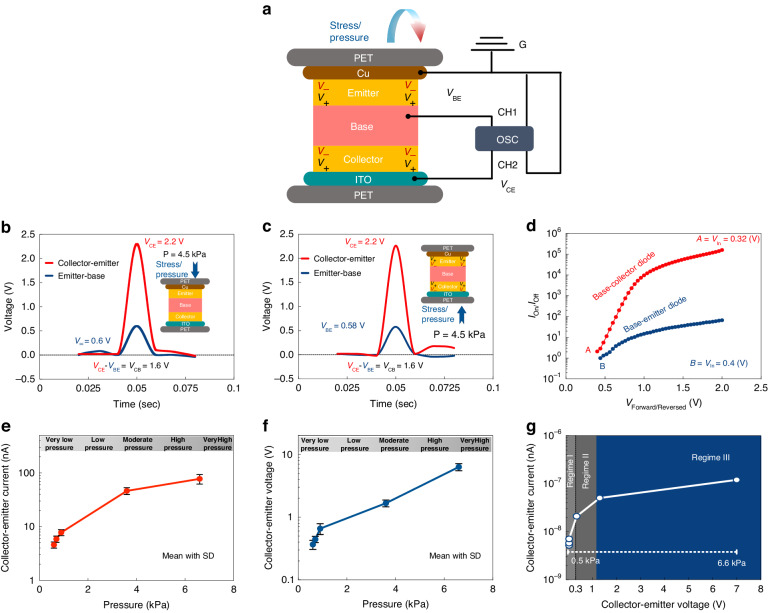
PBJT as a pressure sensor and characteristics in dynamic mode. **a** Pressure sensing mechanism by which the experimental data are extracted. Transient response of the three-terminal n-p-n structure recorded when pressure/stress is applied from (**b**) the top (emitter) and (**c**) the bottom (collector). The applied pressure is kept constant at 4.5 kPa through the utilization of a pressure cell. **d** I_On/iOff_ for the corresponding the base-collector (static reversed biased) and the base-emitter (static forward biased) diodes versus the absolute voltage (reversed/forward). Dynamic response photograph of the n-p-n structure under various applied pressures with respect to the (**e**) current and (**f**) voltage (mean with SD: mean values with the standard deviation). **g** dynamic response of the device under applied pressure showing distinct functional regimes

Any mechanical stress/pressure modulates the PBJT which consequently drives the device in a dynamic mode. The dynamic response of the device differs from the traditional static behavior of a BJT, where the device is statically biased through the base. Interconnection of the dynamic and static response of the device is addressed to clarify how the PBJT functions. The experimental design layout, in which the PBJT is used to extract the data in pressure sensing, is shown in Fig. 4a. Figure 4b-c shows the transient response of the three-terminal device in the common emitter configuration for the base-emitter, and base-collector diodes versus the collector-emitter output signal under a particular applied pressure direction and over a specific area of exertion. Both *n*-type ZnO layers are symmetric, and the only difference between the base-collector and the base-emitter diodes is the impedance in each junction diode due to reversal of the deposition sequences. Therefore, the collector-base voltage accounts for 70% of the output signal (collector-emitter), while the piezotronic base-emitter diode covers 30%. This phenomenon is to be related to the interface states of the emitter-base p-n diode where the ZnO is sputtered on the polymer base. The interface states in vertically-stacked structures are always the prominent factors in deterioration of the device characteristics and operational range.

The PBJT is capable of rectification via various routes depending upon the polarization direction. Figure 4d presents the rectification ratio, (R.R) of the base-emitter and the base-collector diodes individually. The base-collector diode has a higher R.R. than the base-emitter diode which is believed to be related to the interface issue of the ZnO semiconductor deposited on top of the P3HT polymer as well. The use of the n-p-n structure is justified when it is perceived that due to any mechanical stress/pressure, the reversed p-n diode amplifies and regulates the signal through modulation by the generated piezoelectric potential. Additionally, the forward-biased diode is taken to enhance the signal, playing a role in attaining higher sensitivity as a sensing unit. To investigate the device considering two separate base-emitter and base-collector diodes, a detailed study is given in the Supplementary Information (Figs. S4–1). The output characteristics of the emitter-base and collector-base diodes along with the working mechanism under any stress applied from various directions are given in Figs. S4–1a–f.

**Fig. 5:**
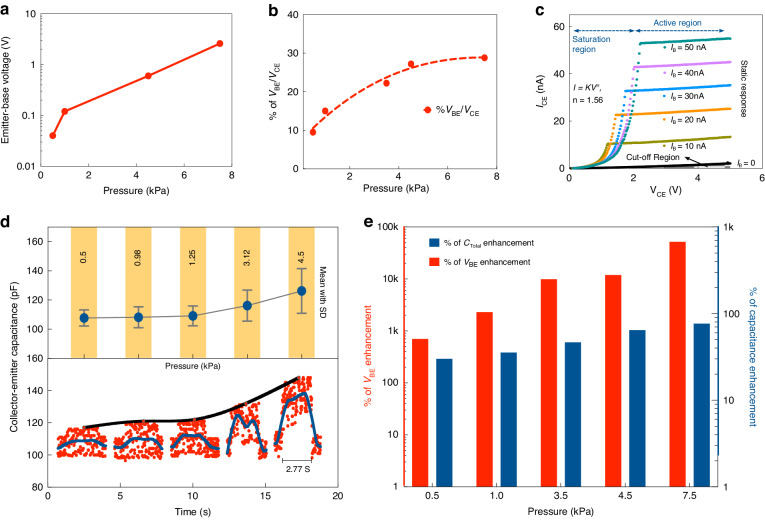
Analysis of the base-emitter junction in transient mode, the output characteristics of the PBJT in the static state, and static pressure detection. **a** Base-emitter voltage versus applied stimuli. **b** V_BE_/V_CE_ ratio under applied pressure. **c** Output characteristics of the three-terminal piezotronic BJT under the common emitter configuration. **d** Variation trend of the collector-emitter system capacitance under applied static pressures ranging from 0.5 kPa to 4.5 kPa. (On the top, mean values with the standard deviation derived and plotted for reasonable sampling rates under various applied pressure). **e** Percentage of ${V}_{{BE}}$ enhancement and percentage of system capacitance growth under dynamic/static applied pressure

Figure 4e, f show the dynamic response of the device corresponding to the current and voltage respectively, under various applied mechanical pressures ranging from very low (0.6 kPa) to moderate (~2.2 kPa) and very high (~10 kPa) over an area of 1 cm × 1 cm. For each pressure point, three values, namely, the maximum, minimum and average, are shown to demonstrate the accuracy of the device behavior. The transient response of the PBJT is presented from a dynamic standpoint, (Fig. 4g). Three distinct regimes are shown that are associated with the device in the “OFF-state, “ON state” and saturation mode. As long as ${V}_{{BE}} < {V}_{{BEON}}$, ie., the “turn-on” voltage is not reached, the base-emitter junction is off; consequently, injection of electrons from the emitter to the base is weak (regime I) and the device is off. The obtained signal is still amplified and superior to that of other piezotronic-based devices (such as: ITO/ZnO/Cu and ITO/ZnO/P3HT/Cu) due to the decrease in the overall built-in capacitance $\left(\frac{d{V}_{{CE}}\left(t\right)}{{dt}}=\frac{d(\frac{Q(t)}{C(t)})}{{dt}}\right)$. As ${V}_{{BE}}$ develops such that ${V}_{{BE}}={V}_{{BEON}}$ ie., the “turn-on voltage” is reached, the base-emitter junction will be turned on; consequently, injection of induced piezoelectric charges from the emitter to the base occurs. In this scenario, the device works in the saturation region (regime II), where the signal is amplified due to the injection of electrons into the base and their effective sequential sweeping toward the collector. The transient current, which has a displacement current component (due to the base-emitter and collector-base depletion regions), and the additional conduction current component (due to the transient bipolar action that occurs as the base-emitter junction turns on) amplifies the transient current response. Increasing the applied pressure leads to ${V}_{{CE}}$ growth such that it satisfies ${V}_{{BE}}\gg {V}_{{BEON}}$, ie., the “turn-on voltage” is exceeded, where more electrons are injected from the emitter into the base and consequently, sweept to the collector, resulting in ${V}_{{CE}}$ enhancement. In this case, the device works as a BJT in the active region (regime III). Figure 5a presents the emitter-base voltage versus the applied pressure. Enhancement of the base-emitter voltage due to the induced piezoelectric potential can be achieved (Figs. S4–2). An increase in the applied pressure results in more charge being injected from the emitter to the base and consequently, enhances the collector-emitter voltage and current. This justifies the “control” of the base through applied pressure in the dynamic state.

Enhancement in piezoelectric charge generation and charge injection into the base increases the base-emitter voltage. It was previously shown that the collector-emitter voltage consists of base-emitter and base-collector voltages that are coupled due to the PBJT structure (two piezotronic ZnO layers on the emitter and collector sides). Figure 5b shows the base-emitter voltage/collector-emitter voltage ratio, which supports the working mechanism of the PBJT in the dynamic state. An increase in applied pressure leads to enhancement of base-emitter voltage / collector-emitter voltage ratio. Hence, as the pressure increases, the charge generation on the emitter side is boosted, and more carriers are injected into the base, which results in base-emitter voltage amplification. Moreover, a larger portion of the open circuit voltage is covered by the base-emitter voltage as the applied pressure increases. This increase ultimately justifies the enhancement in the open-circuit voltage and short-circuit current.

As a transistor-based device, the behavior of the PBJT was verified by extracting the output characteristics in the common emitter configuration and under a static mode (without mechanical stress) as shown in Fig. 5c. The output characteristics of the device shown in Fig. 5c include the output current of the device, (${I}_{{CE}}$), while the collector voltage, ${V}_{{CE}}$, is swept from 0 to 5 V and the base current (${I}_{B}$) is externally biased. It also provides each operational region of the device termed the cut-off, saturation, and active regions. To study the nonlinearity occurring in the saturation region, the parameter *n* as in $I=K{V}^{n}$ was extracted and is given in the inset of Fig. 5c. (for details see the Supplementary Information, Figs. S4–1h).

The PBJT was also simulated for analytical analysis, and the simulation results from electrical and mechanical stand points (Fig. S5) show convergence of the numerical modeling of the device as a piezotronic sensing unit and the data extracted through experiments.

Unlike other piezoelectric-based devices, the PBJT is also capable of static pressure detection as well. It is observed that any static pressure results in a reduction in the semiconductor layer thickness and leads to enhancement of the system capacitance. Since both the emitter and collector are formed by piezoelectric semiconductors, any applied static pressure noticeably increases the system capacitance (Fig. 5d). Figure 5d (top) presents the mean derivation of the plotted data (bottom) along with the standard error bars. In addition to the aforementioned PBJT merits, static pressure detection is a breakthrough in piezoelectric-based devices. As illustrated in Fig. 5d, application of a static pressure for 2.7 s on the device results in a simultaneous increase in the system capacitance. The static pressure detection mode of the PBJT differs from the piezotronic mode, where the transistor is dynamically biased through induced piezoelectric charges injected into the base. In static pressure sensing, the system capacitance variation is the directly extracted signal, while in piezotronic dynamic pressure detection, the voltage/current are the associated outputs. Investigation of the built-in (with a direct relation with the base-emitter junction potential due to charge generation) and system capacitances in dynamic and static pressure detection gives an insight view into two mechanisms of device. Therefore, the percentage increases in the system capacitance and the base-emitter potential under static and dynamic pressure are given in Fig. 5e. This figure shows that the base-emitter potential generated due to induced piezoelectric charges once a dynamic pressure is applied is dominant over the increasing trend of the system capacitance in static pressure detection.

### PBJT as an event-triggering sensor and state-of-the-arts

From a pressure sensing viewpoint, there are wide categories of highly sensitive devices with various mechanisms that have already been studied^37–39^. An examination of piezoelectric-based sensing units implies that these sensors are practically required to be integrated with some CMOS devices for preamplification and interfacing with readout systems. Such systems are termed as piezoelectric-CMOS-based sensors, and encounter several issues such in integration, flexibility, and reliability. Basically, fabrication of a fully flexible device (field effect transistors, organic bipolar transistor, or thin film transistors) with desirable output characteristics is very challenging^40–42^. Therefore, the integration of a flexible piezoelectric-based transducer/sensor with the aforementioned rigid CMOS devices deteriorates the flexibility of the system. Piezoelectric-CMOS-based devices are externally driven, which is challenging for wearable systems. To overcome some of these issues, piezotronic theory has been introduced, which interconnects semiconductors and piezoelectric materials^43^. In this case, several devices have already been reported as piezotronic gate-modulated transistors^44^.

**Fig. 6:**
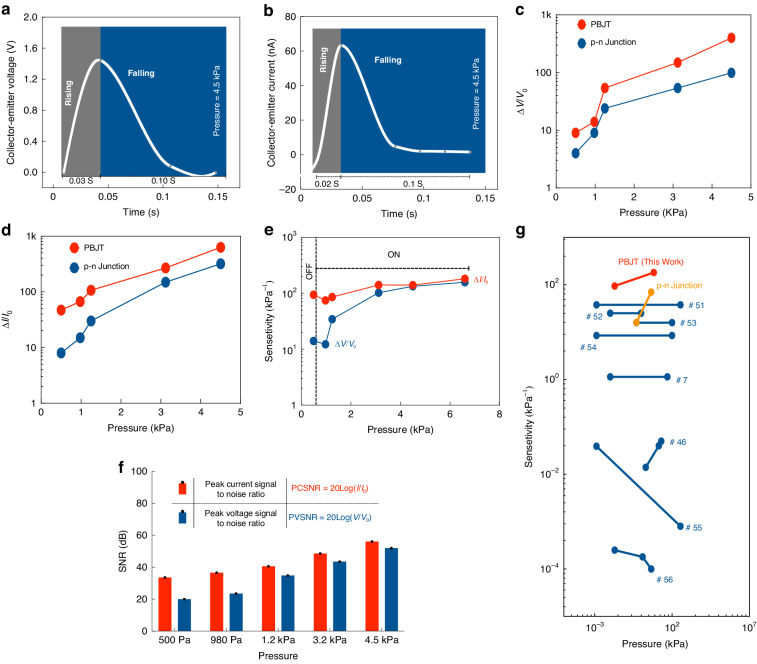
Experimental analysis of the device as a sensing unit. Transient response of the PBJT with respect to the (**a**) collector-emitter voltage and (**b**) collector-emitter current. **c** $\triangle V/{V}_{0}$ and (**d**) $\triangle I/{I}_{0}$ versus applied pressure as for the PBJT and a p-n junction diode. **e** Sensitivity of the PBJT based on the voltage and current. **f** Peak current/voltage signal-to-noise-ratio. **g** Comparison among state-of-the-art devices and the PBJT in terms of sensitivity and showcasing superiority of the proposed piezotronic bipolar junction transistor

Here, the device was experimentally evaluated under low-frequency dynamic stimuli to investigate its sensitivity as a sensing unit. The magnitude of the signal in sensing units always plays a vital role in determining the extracted “signal-to-noise ratio”. A higher SNR technically avoids the complexity of a sensing system due to the lack of requirement for preamplifiers. Figure 6a-b illustrates the transient voltage/current response of the PBJT in the dynamic state once a pressure of 4.5 kPa is applied towards the emitter. The rising and falling times are 0.03 s–0.1 s, respectively. This finding verifies that the response time to the applied stimuli is quite fast at 0.103 s. The longer falling time corresponds to the change in the collector-emitter capacitance under dynamic pressure. Figure 6c, d show the dynamic response of the device with respect to $\triangle V/{V}_{0}$ and $\triangle I/{I}_{0}$ under a low-frequency dynamic stimulus ranging from 500 Pa to 4.5 kPa respectively. A comparison between the fabricated n-p-n transistor and the p-n junction diode verifies the advantage of the n-p-n transistor in terms of sensitivity. It is evident that the sensitivity in terms of both $\triangle V/{V}_{0}$ and $\triangle I/{I}_{0}$ of the n-p-n device increases as the dynamic pressure slightly grows.

Interestingly, as a hybrid device, the sensitivity of the PBJT described in Fig. 6e is related interestingly to both the current and voltage output signals. In this case, the current-based sensitivity is dramatically high (139.77 kPa^-1^ $[\frac{\triangle {\boldsymbol{I}}/{{\boldsymbol{I}}}_{{\boldsymbol{0}}}}{{\boldsymbol{kPa}}}]$, 88.66 Pa^-1^ $[\frac{\triangle {\boldsymbol{V}}/{{\boldsymbol{V}}}_{{\boldsymbol{0}}}}{{\boldsymbol{kPa}}}]$) and linear, which is highly desirable in practical sensing applications. It also presents two sections corresponding to ON, where the device is on and OFF, where applied pressure is not sufficient to turn on the base-emitter junction.

The SNR in sensing units is relatively low, and it is desirable to decrease the noise through proper grounding or optimizing of the fabrication process of the device. The PBJT provides the signal amplification due to transport modulation that is far greater than that of the noise level. The signal-to-noise ratio for both the current and voltage is given in Fig. 6f. The device in resting mode (where no pressure is applied on the emitter side and there is minimum vibration) gives an almost 2–5 mV equivalent noise voltage signal and a 100 pA equivalent noise current (Fig. S6d, e). The stability and reliability of the PBJT were surveyed (Fig. S6f). Compared to other state-of-the-art sensors, such signal extraction is considered unique and beneficial for further employment in various IoT applications.

The proposed PBJT is compared with state-of-the-art devices in terms of the current sensitivity ($\frac{\triangle {\boldsymbol{I}}/{{\boldsymbol{I}}}_{{\boldsymbol{0}}}}{{\boldsymbol{kPa}}}$) and illustrated in Fig. 6g. The PBJT reaches the highest sensitivities in low-pressure range. Champion sensitivity and linearity of the current-based sensitivity are assumed to be priorities and merits in sensing applications. Table 1 presents a comparison among piezoelectric-based devices, piezotronic diode-based sensors and piezoelectric-modulated field-effect transistors in a schematic way. The sensitivity of the p-n junction diode (tabulated in the second row of Table 1) and its transient response are detailed in the Supplementary Information (Fig. S7).

**Table 1:**
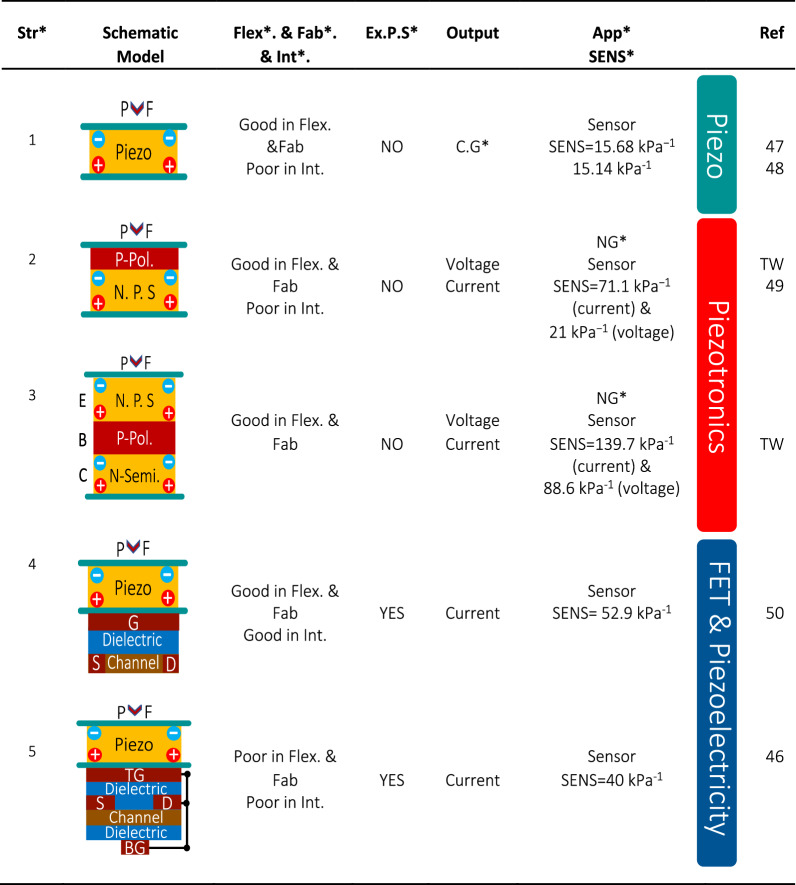
Comparison of piezoelectric modulated FET sensing state-of-the-arts and piezotronic diode-based devices^46–50^

Str* = Structure, Flex* refers to flexibility, Fab* stands for fabrication, Int* implies integration process in terms of circuits and system approach, EX.P.S* = External power source, APP* = Application, SENS* = Sensitivity, Charge generation is abbreviated as C.G*, NG*refers to nanogenerator, TW = This Work

1= Pure piezoelectric material,

2= Piezotronic P-N junction

3= Piezotronic n-p-n transistor

4= Piezoelectric modulated field effect transistor

5= Piezolectric gated dual-gate thin film transistor N. P. S = n-type piezoelectric semiconductor, P-POL. = p-type polymer, P and F are applied pressure or force respectively.

### PBJT pixelated array and practical applications

**Fig. 7:**
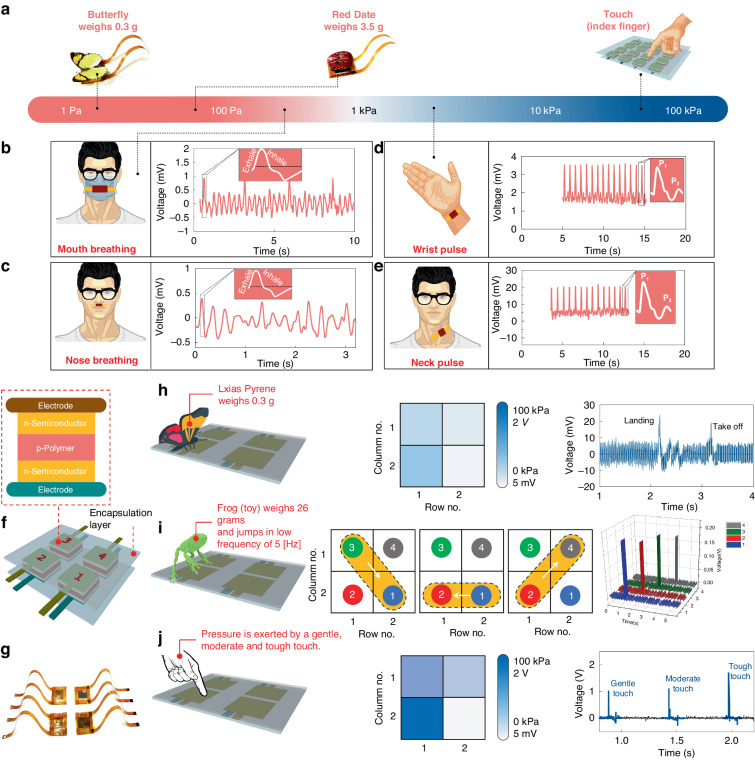
Interactions of the PBJT sensory units/arrays with live/not-live objects. **a** Schematic of wearables and interactions of the device with the environment and humans in sensing applications. Usage of piezotronic n-p-n sensing units for breath detection from the (**b**) mouth, where the sensory unit is mounted on a mask, and (**c**) nose, where the sensory unit is firmly attached to a portion of the upper lips. Pulse recording of a 35-year-old male from the (**d**) wrist and (**e**) neck, and (**f**) a 2$\times$2 array of the PBJT sensor fabricated on a PET substrate, with another supporting layer of PET is placed on top. The array consists of 4 similar pixels in 2 cm$\times$2 cm in size. **g** Photograph of the fabricated array. **h** Landing and taking off states of a particular live butterfly (lxias pyrene) is recorded utilizing an oscilloscope for scanning each row and column. **i** Path detection of a frog toy moving over the sensor array moving with a frequency of 5–10 Hz. **j** Pressure exerted on a unique pixel in an array by gentile, moderate, and high-pressure touch ranging between 4.5 kPa and 10 kPa for a gentle touch by an index finger

To investigate the sensitivity of the sensory unit in practical applications, a single unit along with a 2×2 array of PBJTs was evaluated for particular cases, as described in Fig. 7. First, various interactions based on different pressure ranges are listed in Fig. 7a. A single-pixel PBJT was firmly attached to specific locations on the human body to extract the vital signs given in Fig. 7b–e for mouth breathing, nose breathing, wrist (radial) pulse and neck (carotid) pulse detection. This experiment was performed only to demonstrate the capability of the PBJT for some low-pressure detection to monitor human biological signs. (The extracted data in any form were not utilized for any treatment. The participants in the experiments were four males from whom the authors obtained consent).

A schematic of the pixelated PBJT array with which the experiments were implemented is given in Fig. 7f. A photograph of the fabricated array is also presented in Fig. 7g on which a variety of objects were placed, and the detection signal was recorded. Figure 7h presents the voltage response of the sensor device as a live butterfly (lxias pyrene), weighing 0.3 grams, landed on and took off from a pixel of a pixelated array. The “landing” signal is greater than the “taking off” signal due to the static pressure required to stop the movement while the insect landed. In the “take-off” action, the insect must add a jumping pressure in the opposite direction to achieve the weight release. The aforementioned static force in landing is assumed to be greater than that in jumping (taking off) from a physics stand point, which consequently results in a higher signal in the ‘landing” action.

Figure 7i shows the response of the PBJT as a frog toy weighing 26 grams jumps dynamically jumps on a pixelated sensor area with a frequency of ~5 Hz. All the four pixels in each row and column are simultaneously scanned through the four channels of oscilloscope. The details of the extracted data in this case along with measurements are given in the electrical measurement section. Such signal extraction can be used in principle for path detection or force mapping, as shown.

The crosstalk effect in a pixelated array format of sensors is always an issue, and array layout optimization is challenging. The 3D magnitude of the extracted signal corresponding to each pixel when the toy frog jumped on it was shown to have the least crosstalk effect among the pixels. The data measured through an acquisition board and readout circuit allow conversion of the dynamic stimuli into an image for detection of the real-time movement of an object, which is highly desirable.

Finally, the PBJT was tested for touching action, and the device response under several touching conditions by an index finger, assuming an approximate effective area of 2 cm × 2 cm, is illustrated in Fig. 7j. Three signals corresponding to distinct cases, i.e., low, moderate and high dynamic touch stimuli, are plotted.

Owing to being self-powered and highly sensitive, the PBJT will be triggered as soon as an event associated with mechanical stimuli (pressure) occurs. Therefore, the PBJT is considered as an event-triggered device, which is needed in many applications, such as sound recognition/mapping and sound-to-image conversion for security purposes.

The capability of an event-triggered PBJT with machine learning to be used for artificial intelligence-sound recognition has recently been reported^45^. In addition to the aforementioned applications in which the PBJT function priority has been justified, such an event-triggered, low-cost, autonomous pixelated sensory array with a particular artificial intelligence is of great interest in the prevention of infrastructure (bridges, tunnels, etc.) destruction through structure failure detection and monitoring.

## Discussion

Piezotronics theory, in principle, encompasses semiconductivity and piezoelectricity. Wurtzite materials such as ZnO and GaN are promising candidates for use in piezoelectric processes due to coupling of piezoelectricity with semiconductor properties. The obtained significant current gain even under a very small stress/strain is greater than that of CNT or graphene transistors. In a novel passive hybrid piezotronic bipolar junction transistor (n-p-n) was introduced through the process of charge transport (due to piezoelectric potential generation) across the emitter-base and collector-base junctions. The PBJT appears to be promising for shifting the paradigm in design of the next generation of hybrid NEMS and sensing systems. From the pressure sensing stand point, the PBJT indicates high sensitivity that is competitive with other state-of-the-arts devices. Taking harvesting into account, the PBJT acts well as a nanogenerator, demonstrating its capability as a hybrid device. Hybrid devices, as defined, are of great interest because synergy between energy harvesting and sensing is always challenging. Other key-issues in hybrid devices come from signal amplification (sensing) and rectification (harvesting), which dictate the complexity of such systems. Thus, a unique device such as the PBJT capable of both amplification and rectification simplifies the development of hybrid systems. Moreover, the event-triggered characteristics of the PBJT suggest that its array format is appropriate as an autonomous flexible device for event detection. In addition, ease of fabrication in large-scale electronics gives the priority of PBJT compared to its counterparts.

In this paper, a vertically-stacked sandwich structure of an n-piezoelectric semiconductor/p-semiconductor polymer/n-piezoelectric semiconductor PBJT has been introduced, while the lateral structure based on the specific applications and analysis of the p-n-p design layout remain under study as the future work. The aforementioned PBJT is formed by two piezotronic p-n junction diodes where the emitter and collector are considered as piezoelectric semiconductors. Another design layout that requires further investigation is the piezoelectric p-base/ heavily doped n-semiconductor emitter/ n-semiconductor collector structure. It is assumed that the revolution with the introduction of piezotronics theory will advance the current technology in sensing and harvesting systems.

## Conclusion

In summary, this work provides an in-depth combination of back-to-back piezotronic-based diodes to form a piezotronic bipolar junction transistor, and paves the way for designing self-powered and self-amplified pressure sensors. A PBJT is obtained through the formation of two inorganic-organic heterojunctions. The PBJT can operate as a highly sensitive sensory unit (sensitivity of 139.77 kPa^-1^ $[\frac{\triangle {\boldsymbol{I}}/{{\boldsymbol{I}}}_{{\boldsymbol{0}}}}{{\boldsymbol{kPa}}}]$, and 88.66 kPa^-1^ $[\frac{\triangle {\boldsymbol{V}}/{{\boldsymbol{V}}}_{{\boldsymbol{0}}}}{{\boldsymbol{kPa}}}]$). The proposed device differs from simply placement of two separate p-n junction diodes back-to-back in that the base is common and the two diodes form an integral bipolar transistor. The proposed PBJT is deemed as a hybrid device because it not only benefits from being a passive highly-sensitive pressure sensor, but also acts as an appropriate nanogenerator. Due to the formation of two piezotronic heterojunction diodes, the obtained signal is well-regulated independent of the piezoelectric potential polarization direction. Last but not least, the PBJT provides variations in the input/output signal format with a fast response.

## Materials and methods

### Synthesis of the p-type polymer semiconductor (P3HT)

A 20 mg/ml P3HT solution was prepared with chlorobenzene as the solvent. Magnetic stirring was performed at 50 °C for at least 2 h. After complete dissolution and cooling, the solution was filtered, and then, the P3HT solution was ready for use.

### Fabrication of bottom n-type semiconductor ZnO (collector)

ITO/PET was used as a substrate to deposit the first ZnO layer. The ZnO bulk was RF sputtered while the pressure was maintained at 0.7 ~ 0.77 Pa, and the power was set to 100 W for almost 15 min at room temperature. The aforementioned steps were repeated 8 times to obtain thicknesses ranging from 420–500 nm per the design layout. Finally, the thick ZnO layer was annealed at 100 °C for 30 min.

### Fabrication of piezotronic bipolar junction transistors

ZnO/ITO/PET was used as a substrate to coat 100 μl of a p-type polymer (P3HT) on top. P3HT was doctor bladed to evenly spread the polymer and form a uniform layer. The device was annealed at 70 ~ 80 °C for 15 min to volatilize the solvent. A second ZnO layer was deposited through RF sputtering with the same methods and conditions as those used for the bottom ZnO layer. The device was annealed at 100°C for >30 min to obtain the 002 orientation of wurtzite crystallization of ZnO.

### Fabrication of p-n junction diode

An RF sputtering process was performed at room temperature to form a ZnO layer on a PET substrate. Then, the device was annealed at 100 °C to optimize the crystallization of the piezoelectric bulk ZnO. In the next step, a 1 µm-thick p-type polymer (P3HT) was coated on top to form the p-n junction structure. Finally, the top electrode was patterned on top of P3HT.

### Material characterization

The morphology of the fabricated n-p-n structure was examined through SEM/TEM (Carl Zeiss (Germany) ZEISS SUPRA® 55, JEOL (Japan), and JEM-3200FS), and X-ray diffraction of each ZnO layer was performed using a Bruker (Germany) D8 Advance with a Cu target, a voltage ≤ 40 kV, and a current ≤ 40 mA.

### Electrical measurements of the PBJT

A low-noise multimeter (KEITHLEY, DMM6500) and an OSC (Tektronix, MDO3054) were utilized to extract the output signal of the device in the form of a current or a voltage. The transistor device was characterized with a probe station Keysight B1500A and an EVERBEING (Taiwan, China). A live Ixias pyrene butterfly was used in the chamber in which the array of devices was placed to detect flying object interactions. A toy frog weighing 26 grams that jumped at a frequency of 5–10 Hz was used for path detection.

### Supplementary information

Supplemental Material

## References

[1] Tyler RR (2019). A. Bio-Integrated Wearable Systems: A Comprehensive Review. Chem. Rev..

[2] Song Y (2020). Wireless battery-free wearable sweat sensor powered by human motion. Sci. Adv..

[3] Wang B (2022). Wearable aptamer-field-effect transistor sensing system for noninvasive cortisol monitoring. Sci. Adv..

[4] Lee S (2020). Nanomesh pressure sensor for monitoring finger manipulation without sensory interference. Science.

[5] Oh YS (2021). Battery-free, wireless soft sensors for continuous multi-site measurements of pressure and temperature from patients at risk for pressure injuries. Nat. Commun..

[6] Yu Q (2022). Highly sensitive strain sensors based on piezotronic tunnelling junction. Nat. Commun..

[7] Gong S (2014). A wearable and highly sensitive pressure sensor with ultrathin gold nanowires. Nat. Commun..

[8] Chen M (2019). An ultrahigh resolution pressure sensor based on percolative metal nanoparticle arrays. Nat. Commun..

[9] An BW (2018). Transparent and flexible fingerprint sensor array with multiplexed detection of tactile pressure and skin temperature. Nat. Commun..

[10] Yan W (2021). Giant gauge factor of Van der Waals material-based strain sensors. Nat. Commun..

[11] Li H (2020). A Supersensitive, Multidimensional Flexible Strain Gauge Sensor Based on Ag/PDMS for Human Activities Monitoring. Sci. Rep..

[12] Araromi OA (2020). Ultra-sensitive and resilient compliant strain gauges for soft machines. Nature.

[13] Wang J (2016). Sustainably powering wearable electronics solely by biomechanical energy. Nat. Commun..

[14] Li J (2021). Body-coupled power transmission and energy harvesting. Nat. Electron..

[15] Jinno H (2021). Self-powered ultraflexible photonic skin for continuous bio-signal detection via air-operation-stable polymer light-emitting diodes. Nat. Commun..

[16] Wu W (2016). Piezotronics and piezo-phototronics for adaptive electronics and optoelectronics. Nat. Rev. Mater..

[17] Zong X (2017). Zinc oxide nanorod field effect transistor for long-time cellular force measurement. Sci. Rep..

[18] Wang X (2019). Van der Waals negative capacitance transistors. Nat. Commun..

[19] Wang L (2021). Advances in piezotronic transistors and piezotronics. Nano Today.

[20] Zhang Y (2011). Fundamental theory of piezotronics. Adv. Mater..

[21] Kumar R (2021). Review on ZnO-based piezotronics and piezoelectric nanogenerators: aspects of piezopotential and screening effect. J. Phys. Mater..

[22] Zhao Z (2021). Selection rules of triboelectric materials for direct-current triboelectric nanogenerator. Nat. Commun..

[23] Tan C (2020). A high-performance wearable strain sensor with advanced thermal management for motion monitoring. Nat. Commun..

[24] An C (2021). Piezotronic and piezo-phototronic effects of atomically-thin ZnO nanosheets. Nano Energy.

[25] Zhao ZH (2015). Piezotronic Effect in Polarity-Controlled GaN Nanowires. ACS Nano.

[26] Tangi M (2018). Observation of piezotronic and piezo-phototronic effects in n-InGaN nanowires/Ti grown by molecular beam epitaxy. Nano Energy.

[27] Puneetha P (2021). Temperature dependence of the piezotronic effect in CdS nanospheres. Nano Energy.

[28] Wu W (2014). Piezoelectricity of single-atomic-layer MoS2 for energy conversion and piezotronics. Nature.

[29] Xi F (2018). Tribotronic bipolar junction transistor for mechanical frequency monitoring and use as touch switch. Microsyst. Nanoeng..

[30] Iranmanesh E (2023). Piezotronic Bipolar Junction Transistor as a Wearable Energy Harvester. IEEE Trans. Electron Devices.

[31] Nour ES (2017). Zinc oxide piezoelectric nano-generators for low frequency applications. Semicond. Sci. Technol..

[32] Gardeniers H (1998). Preferred orientation and piezoelectricity in sputtered ZnO. films. J. Appl. Phys..

[33] Ellmer K (2016). Intrinsic and extrinsic doping of ZnO and ZnO alloys. J. Phys. D: Appl. Phys..

[34] Sze, S. M. & Ng, K. K. *Physics of Semiconductor Devices* 3rd edn, 832 (John Wiley & Sons, New Jersey, 2007).

[35] Zhu P (2018). Ultra-high sensitivity strain sensor based on piezotronic bipolar transistor. Nano Energy.

[36] Yang J (1999). An analytical model for the saturation characteristics of bipolar-mode static induction transistors. Solid- State Elect..

[37] Qin R (2021). A new strategy for the fabrication of a flexible and highly sensitive capacitive pressure sensor. Microsyst. Nanoeng..

[38] Pang C (2012). A flexible and highly sensitive strain-gauge sensor using reversible interlocking of nanofibres. Nat. Mater..

[39] Ma Y (2017). A highly flexible and sensitive piezoresistive sensor based on MXene with greatly changed interlayer distances. Nat. Commun..

[40] Li N (2020). Large-scale flexible and transparent electronics based on monolayer molybdenum disulfide field-effect transistors. Nat. Electron..

[41] Leydecker T (2016). Flexible non-volatile optical memory thin-film transistor device with over 256 distinct levels based on an organic bicomponent blend. Nat. Nanotech..

[42] Wang SJ (2022). Organic bipolar transistors. Nature.

[43] Liu Y (2015). Fundamental theories of piezotronics and piezo-phototronics. Nano Energy.

[44] Ge R (2023). Dual-modal piezotronic transistor for highly sensitive vertical force sensing and lateral strain sensing. Nat. Commun..

[45] Iranmanesh E (2023). Event-Trigger Dual-Signal-Mode Flexible Piezotronic Bipolar Junction Transistor with Machine Learning for AI-Sound Recognition. IEEE Electron Device Lett..

[46] Li W (2020). A Force and Temperature Sensor Array Based on 3-D Field-Coupled Thin-Film Transistors for Tactile Intelligence. IEEE Trans. Elect. Dev..

[47] Luo J (2023). Highly sensitive, wide-pressure and low-frequency characterized pressure sensor based on piezoresistivepiezoelectric coupling effects in porous wood. Carbohydr. Polym..

[48] Luo J (2021). Flexible piezoelectric pressure sensor with high sensitivity for electronic skin using near-field electrohydrodynamic direct-writing method. Extrem. Mech. Lett..

[49] Lee K (2012). P-Type Polymer-Hybridized High-Performance Piezoelectric Nanogenerator. Nano. lett..

[50] Wang F (2020). Piezopotential gated two-dimensional InSe field-effect transistor for designing a pressure sensor based on piezotronic effect. Nano Energy.

[51] Lee Y (2018). Flexible Ferroelectric Sensors with Ultrahigh Pressure Sensitivity and Linear Response over Exceptionally Broad Pressure Range. ACS Nano.

[52] Pang Y (2018). Epidermis Microstructure Inspired Graphene Pressure Sensor with Random Distributed Spinosum for High Sensitivity and Large Linearity. ACS Nano.

[53] Luo N (2017). Hollow-Structured Graphene−Silicone-Composite-Based Piezoresistive Sensors: Decoupled Property Tuning and Bending Reliability. Adv. Mater..

[54] Bae GY (2016). Linearly and Highly Pressure-Sensitive Electronic Skin Based on a Bioinspired Hierarchical Structural Array. Adv. Mater..

[55] Kim SY (2015). Highly Sensitive and Multimodal All-Carbon Skin Sensors Capable of Simultaneously Detecting Tactile and Biological Stimuli. Adv. Mater..

[56] Park H (2015). Stretchable Array of Highly Sensitive Pressure Sensors Consisting of Polyaniline Nanofibers and Au-Coated Polydimethylsiloxane Micropillars. ACS Nano.

