# Supplementary material for: Organic-inorganic hybrid piezotronic bipolar junction transistor for pressure sensing
Source: Microsyst Nanoeng. 2024 Jun 20;10:80. doi: 10.1038/s41378-024-00699-0 (PMC11189938; doi:10.1038/s41378-024-00699-0)
Supplement: Supplementary file 1 — Supplemental Material [file 41378_2024_699_MOESM1_ESM.pdf]

Organic-Inorganic Hybrid Piezotronic Bipolar Junction Transistor for Pressure Sensing

Emad Iranmanesh[1,2], Zihao Liang[1], Weiwei Li[3], Congwei Liao[1], Shunyu Jin[4], Chuan Liu[5], Kai Wang[5], Shengdong Zhang[1], Charalampos Doumanidis[2,6], Gehan A. J. Amaratunga[7], Hang Zhou[1]

[1]School of Electronic and Computer Engineering, Peking University, Shenzhen Graduate School, Shenzhen, 518055, P. R. China.

[2]School of Mechanical Engineering, Guangdong Technion-Israel Institute of Technology, Shantou, 515063, P. R. China.

[3]State Key Laboratory of Microelectronics Device and Integrated Technology, Institute of Microelectronics, Chinese Academy of Sciences, Beijing 100029, P. R. China.

[4]Hefei National Research Center for Physical Sciences at the Microscale, University of Science and Technology of China, Hefei, 23000, PR China

[5]School of Electronics and Information Technology, Sun Yat-sen University, No. 132 East Waihuan Road, Guangzhou, 510006, P. R. China.

[6]Department of Mechanical, Aerospace, and Biomedical Engineering, University of South Alabama, Shelby Hall, 3128 Mobile, AL 36688, United States.

[7]Electrical Engineering Division, Department of Engineering, University of Cambridge, 9 JJ Thomson Avenue, Cambridge, CB3 0FA, United Kingdom, and Zhejiang University, International Campus, Haining, China.

Correspondence authors: Emad Iranmanesh: (iranmanesh.em@gmail.com) or Hang Zhou (zhouh81@pkusz.edu.cn) or Kai Wang (wangkai23@mail.sysu.edu.cn).

Supporting Information:

This additional file includes:

1. Fabrication process of PBJT and piezotronic p-n junction
2. Energy band diagram and working mechanism of p-n junction diode
3. Numerical modeling and theory
4. Piezotronic n-p-n transistor as two diodes back-to-back
5. Simulation of PBJT as a sensory unit
6. Details of experimental data extracted by PBJT as a pressure sensor
7. Details of experimental data extracted by p-n junction diode as a pressure sensor

References

Supplementary Information S1: Fabrication process of the PBJT

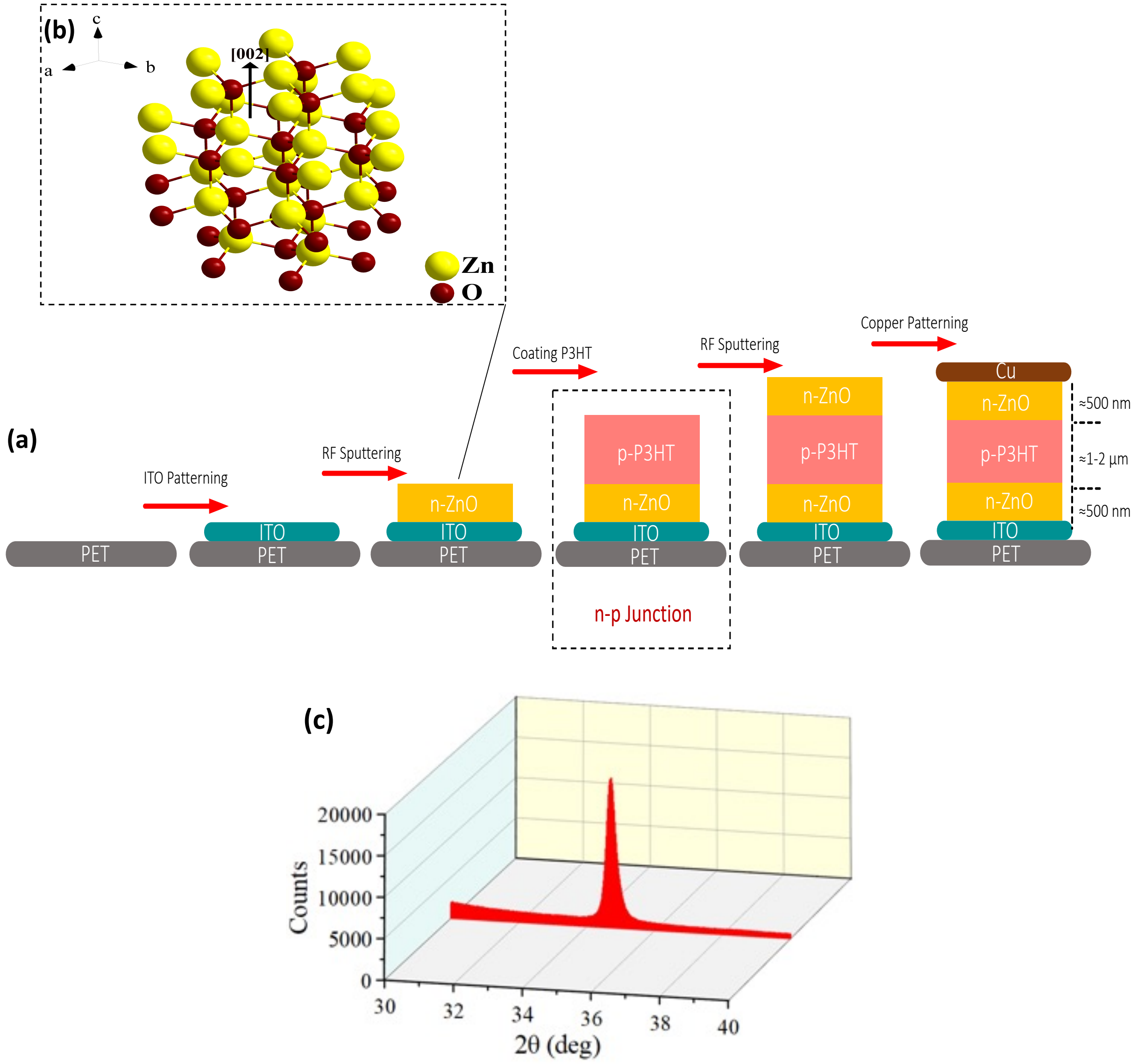

Fig. S1. Device fabrication of piezotronic bipolar junction transistor a) n-p-n structure sandwiching p-type polymer by two n-type piezoelectric semiconductor materials and p-n junction diode along with thickness differentiation between layers. **b)** Crystal orientations of ZnO semiconductor layers **c)** XRD image of p-n junction showing the orientation of wurtzite crystals of ZnO.

Piezotronic n-p-n bipolar junction transistor is deemed to form by placing two piezotronic diodes back-to-back in theory. Therefore, a comparison between n-p and p-n junction heterostructures are

focused. The fabrication process is detailed as: firstly, an ITO bottom electrode is patterned on a flexible PET substrate and then a 550nm thick ZnO dielectric semiconductor as n-type piezoelectric semiconductor material has been RF sputtered. P3HT as p type polymer is spin coated atop to form a diode-like p-n junction. P3HT might be replaced by the nominated materials as conjugated polymers in this scenario[1-4] such as: PEDOT:PSS, PMMA, PTAA, Nickel oxide. This process continues by sputtering another n-type piezoelectric semiconductor layer (ZnO) atop the p-type hole transport layer. Ultimately, a copper electrode is patterned atop to form the two-terminal device. Piezoelectric characteristics of ZnO semiconductor has been introduced decades ago where any mechanical stimuli exerted on ZnO surface generates piezoelectric potential due to polarization theory. Researchers have focused on optimization piezoelectricity and fabrication process of ZnO semiconductor films[5-8].

The piezotronic bipolar transistor is formed once the two p-n and n-p diodes are placed back-to-back as the base is in common. Technically, two discrete p-n and n-p diodes will not form an n-p-n transistor. These two diodes in the given PBJT are similar in size with different behaviors. Therefore, we better have a look on the fabrication difference among them which supports variations in piezoelectric modulation in practical terms. Figure S1a describes the difference in fabrication process of these two heterojunction diodes. It ascribes the fabrication of n-p diode where n-type semiconductor is deposited and the p-type polymer coated atop. Bonding of Zn and oxygen atoms to form the required orientation is shown in Fig. S1b. Conversely to the given step, to form the p-n diode structure, only n-type semiconductor is deposited atop the p-type polymer. As expected, interface issues always in heterostructures prevails and overcoming such issues are considered to be very challenging. Therefore, the interface connection between the bottom n-type semiconductor and p-type polymer is well-formed while the interface between the top ZnO layer

with P3HT may accompany with some surface defects which consequently affects the performance of the device. Crystal orientation of piezotronic ZnO layer in n-p junction diode is investigated through XRD diffraction analysis (Fig. S1c).

Supplementary Information S2: Energy band diagram and working mechanism of p-n junction diode

Piezotronic n-p-n bipolar junction transistor is assumed prior in terms of pressure sensing compared to a simple n-p heterojunction structure. Figure S2a-b represents the energy band levels of device under resting and applied pressure; as the pressure is applied, electrons flow from copper electrode to the ITO and an equilibrium of state is occurred as shown in Fig S2b. Equivalent electrical circuit of p-n junction is given in Fig. S2c.

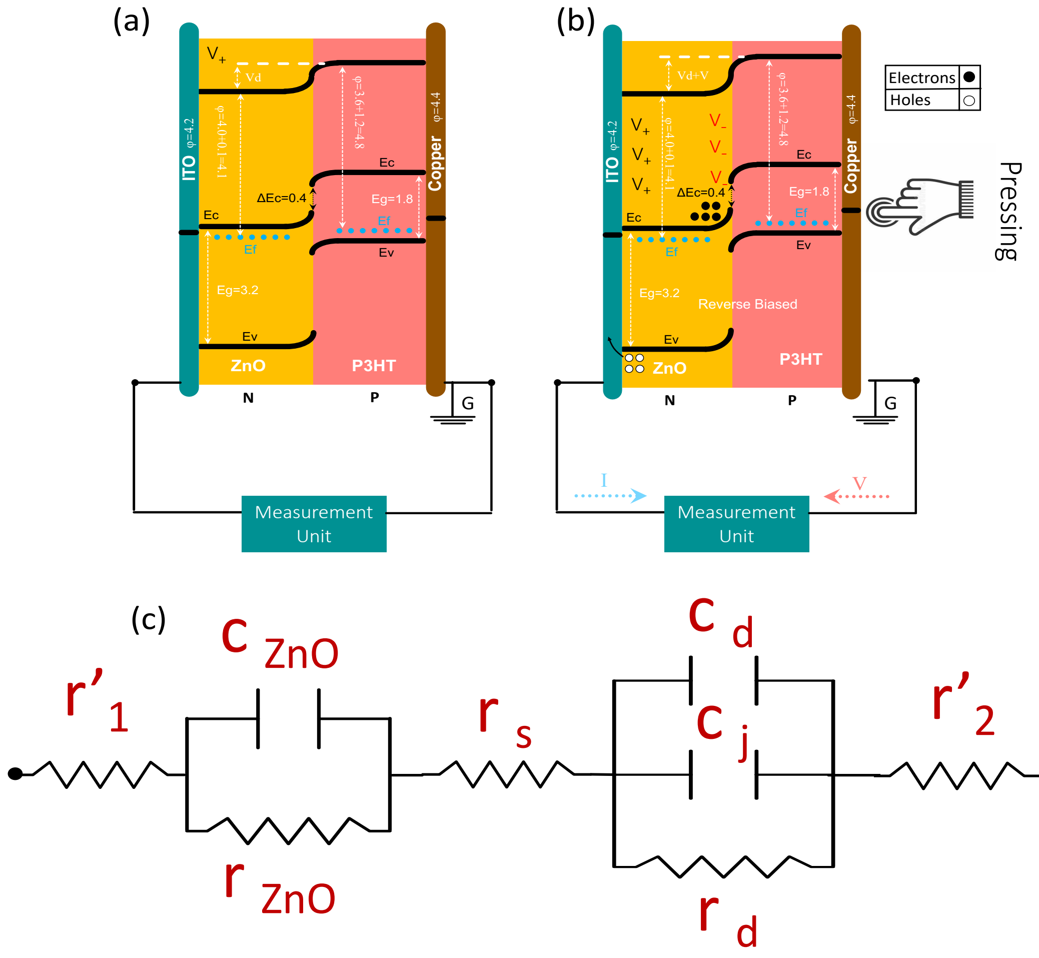

Fig. S2. Energy band diagram of n-p heterojunction structure and equivalent c-r circuit. Schematic illustration of working mechanism of device and the conduction band level a) Under no stress **b)** Under applied stress, **c)** Iconic c-r electrical representation of the n-p heterojunction structure.

Supplementary Information S3: Numerical modeling and theory

In this section a comprehensive modeling is given step by step which provides a complete information about the given theory in the manuscript. It needs to be stated that the modeling is done based on the diffusion theory[9] and therefore, collector current as an output in the given structure can be given as:

$$I_C = \frac{A_E q D_n n_{PB0}}{L_n} Cos\ ech\left(\frac{W_B}{L_n}\right) exp\left(\frac{qV_{BE}}{KT}\right), \tag{1}$$

where A_E is the cross-sectional area of emitter-base junction and absolute value of the unit electronic charge is given by q. W_B is the width of base region, n_{PB0} is thermal equilibrium electron concentration in p-type semiconductor. $L_n = \sqrt{D_n \tau_n}$ is the diffusion length of holes and D_n is considered as diffusion coefficient for electrons. K is the Boltzmann factor and T is the temperature in Kelvin. V_{BE} is base-emitter junction voltage.

$$V_{CE} = V_C - V_B + V_B - V_E, \tag{2}$$

V_{CE} as the collector-emitter voltage given in (2) can be modeled in terms of generated piezoelectric charges and in-series capacitances of collector-base and emitter-base junctions.

$$V_{CE} = \frac{\left(d_{33}.F_{33} + \left(\frac{l}{t}\right)d_{31}.F_{31}\right)\left(C_{C-B} + C_{E-B}\right)}{C_{C-B}.C_{E-B}}, \tag{3}$$

The total generated charges is given as:

$$Q_{Total} = d_{33}.F_{33} + \left(\frac{l}{t}\right)d_{31}.F_{31}, \tag{4}$$

where d_{33} and d_{31} are the piezoelectric constants in 33 and 31 directions, respectively. F is the applied force; l and t are the length and thickness of the collector/emitter layers respectively.

Due to formation of depletion region at emitter-base and collector-base p-n junctions, $C_{p\text{-}n(C\text{-}B)}$ and $C_{p\text{-}n(E\text{-}B)}$ are introduced. The PBJT as an energy harvester is theoretically defined as two p-n junction diodes back-to-back. In an ideal case, the capacitance in each collector-base and emitter-base junctions are as:

$$C_{Collector-Base} = \frac{C_{j(C-B)}.C_{ZnO(Collector)}}{C_{j(C-B)} + C_{ZnO(Collector)}}, \tag{5}$$

$$C_{Emitter-Base} = \frac{C_{j(E-B)}.C_{ZnO(Emitter)}}{C_{j(E-B)} + C_{ZnO(Emitter)}}, \tag{6}$$

where capacitances of collector-base, emitter-base junctions and ZnO bulk are placed in series. $C_{J(C\text{-}B)}$ refers to capacitance junction at collector-base, and $C_{J(E\text{-}B)}$ defines as capacitance forms at emitter-base junction. $C_{ZnO\ (Emitter)}$ and $C_{ZnO\ (Collector)}$ are bulk ZnO capacitances at emitter and collector sides respectively.

Therefore, the collector current is given as:

$$I_{CE} = \frac{A_E q D_n n_{PB0}}{L_n} Cos\,ech\left(\frac{W_B}{L_n}\right) exp\left\{\left(\frac{q.\left(\frac{\left(d_{33}.F_{33} + \left(\frac{l}{t}\right) d_{31}.F_{31}\right)\left(C_{j\,(E-B)} + C_{ZnO(Emitter)}\right)}{C_{j\,(E-B)}.C_{ZnO(Emitter)}}\right)}{KT}\right)\right\}, \tag{7}$$

$C_{Emitter\text{-}\ Base}$ is the formed capacitances related to emitter-base junction. Apart from amplified current in response to the dynamic stimuli (eq. 7), due to re-distribution of polarized charges and in-series capacitance effect, voltage signal also gets boosted (eq. 3). The enhanced V_{CE} is well-suited for pressure level sensing experiment needless of any extra circuitry for signal conversion.

Based on the given model voltage sensitivity of device can be extracted as:

$$S = \frac{V_{CE}}{F_{33}} = \frac{\left(\frac{Q\left(C_{C-B} + C_{E-B}\right)}{C_{C-B}.C_{E-B}}\right)}{F_{33}}, \tag{6}$$

Relation between generated charge in each region and the applied stress results in:

Current sensitivity of device based on the I_C and applied stress:

$$R = \frac{I_C}{F_{33}}, \tag{7}$$

Output power of device is also extracted as:

$$P_{Output} = V_{CE} . I_{CE}, \tag{8}$$

$$d_{31} = \frac{e_{31}}{C_{13}}, \tag{9}$$

$$d_{33} = \frac{e_{33}}{C_{33}}, \tag{10}$$

d_{33} and d_{31} are assumed to be the piezoelectric modulus of n-type semiconductor. e_{33} and e_{31} are the piezoelectric constant of piezoelectric material and C_{13}, C_{33} elastic constant of ZnO.

Supplementary Information S4: Piezotronic n-p-n transistor as two diodes back-to-back

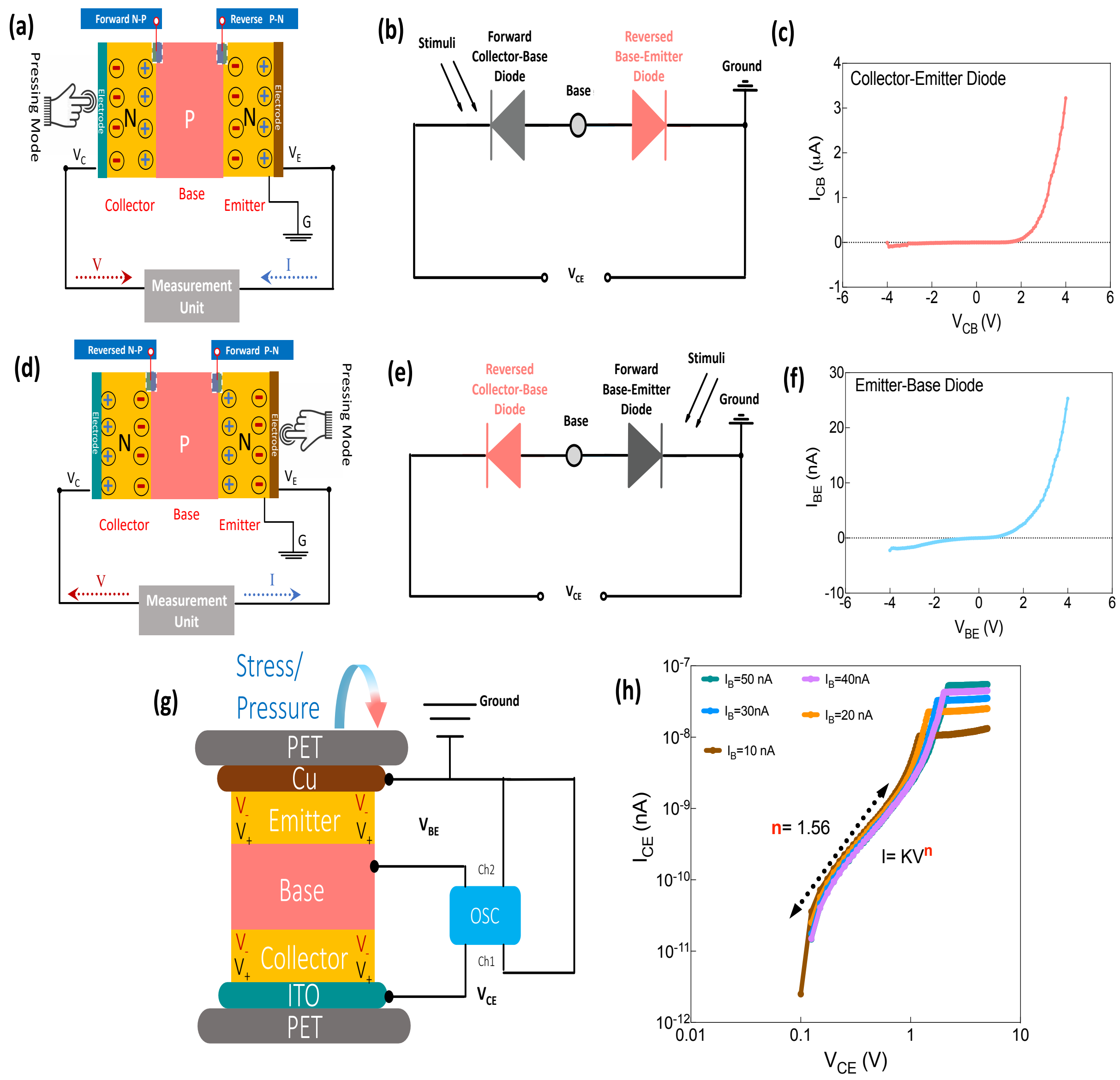

Fig. S4-1. Piezotronic n-p-n transistor as two diodes back-to-back. a) Schematic of device as pressure is applied under collector, **b)** Electrical equivalent circuit once the emitter-base diode is reversed (red) and collector-base is forward (black). **c)** I-V curve of collector-base p-n junction diode. **d)** Piezotronic n-p-n transistor under pressure applying towards emitter, **e)** Equivalent circuit showing collector-base diode as in forward mode and emitter-base in reversed (red). **f)** Characteristics of emitter-base diode as statically biased and voltage is sweeping from -4 (V) to 4 (V). **g)** Schematic design layout of experiment by which data have been extracted for Fig. 3b and 3c. **h)** Extraction of n=1.56 as in $LogI=K+nLogV$ by Curve fitting based on the output characteristics of BJT (Fig. 5c)

Piezotronic n-p-n transistor can be modeled as two diodes are taken into account back-to-back from circuit stand point of view. Therefore, in this case, an investigation of two diodes formed as in collector-base and emitter base are both considered. Piezotronic theory in p-n junction has already been studied in some particular applications rather than sensing[10]. As pressure is applied on emitter, aforementioned two diodes are modulated through induced piezoelectric potential to be in reversed and forward state depending on polarization direction. As the pressure is applied towards collector, emitter-base diode acts as a reversed p-n junction whereas collector-base n-p diode functions in forward state (Fig. S4a-b). Fig. S4c illustrates the characteristics of collector-base diode as it is biased statically through an external source and the voltage is swept from -4 V to + 4 V. Likely, as the pressure is applied towards the emitter (shown in Fig. S4d and S4e), the emitter-base diode characteristics is given in Fig. S4f.

To investigate the nonlinearity occurs in saturation region of transistor characteristics curve given in Fig. 5c, space charge limit theory is addressed. Therefore, the value of n in $I = V^n$, can be rewritten as: $LogI = K + nLogV$. Finally, n is extracted as 1.56 through curve fitting and showed in Fig. S4h.

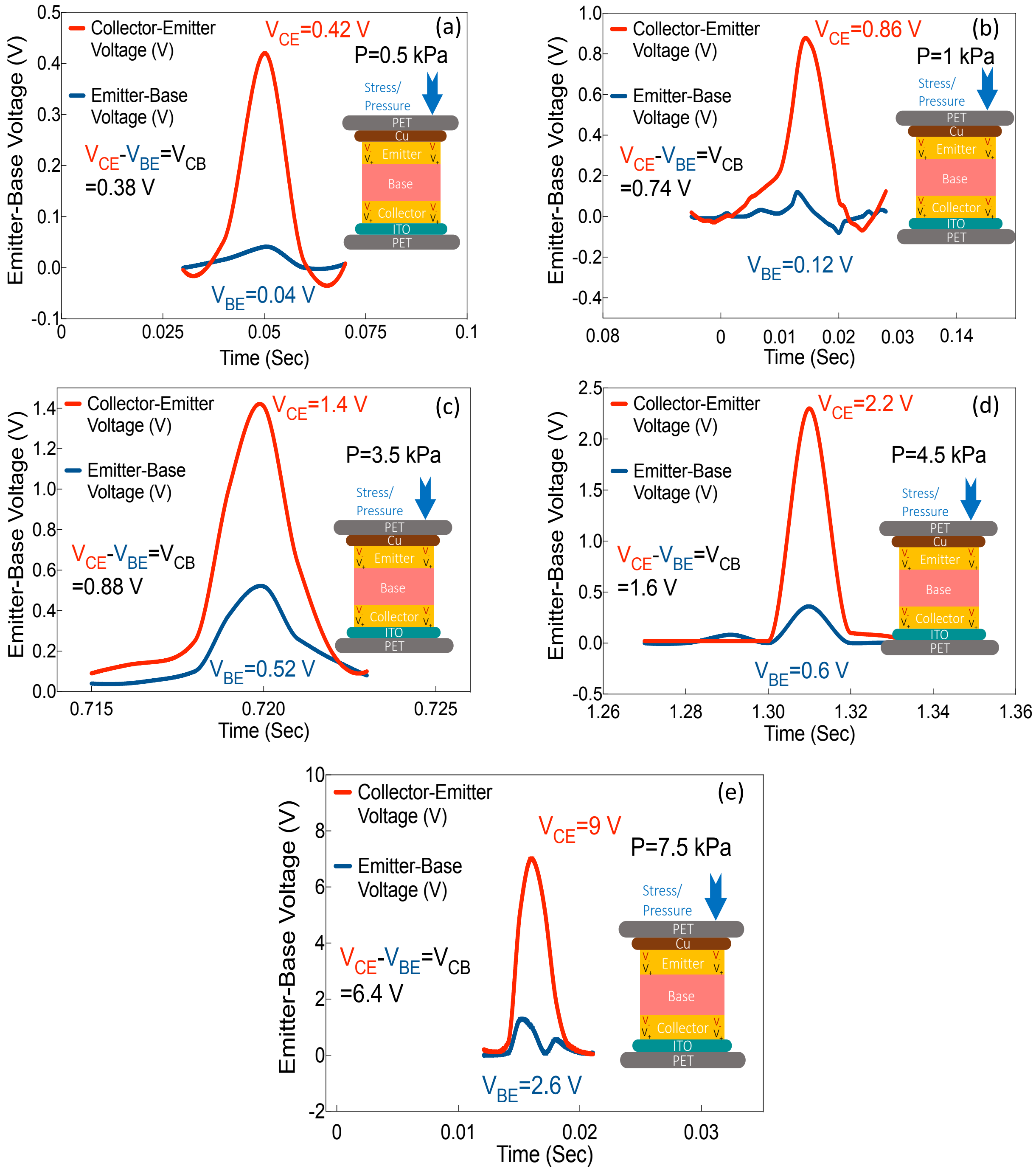

Fig. S4-2. Control over the base in PBJT dynamic state under applied pressure. Schematic of device under applied pressure which reaches to **a)** 0.5 kPa, **b)** 1 kPa, **c)** 3.5 kPa, **d)** 4.5 kPa, and **e)** 7.5 kPa

Figure S4-2 shows the extracted base-emitter and collector-emitter voltages simultaneously various applied pressure. As the pressure is applied on emitter side, both emitter and collector will get affected and piezoelectric charge will be generated. If the generated charge will be enough to

turn on the heterojunctions, $V_{CE}(t)$ is observed and this bipolar action results in transient current of $I_{CE}(t)$ which has the displacement current due to formation of collector-base and base-emitter depletion regions.

It can be seen from these results firstly, the charge generation due to any applied stress on emitter side occurs due to piezoelectricity behavior of emitter. Besides, considering PBJT as two p-n junctions back-to-back, as the pressure is applied from emitter side, the base-emitter junction diode is forward and the base-collector diode is reversed. Depletion width in base-emitter due to forward biased, is narrower and if the obtained V_{BE} is greater than the "turn-on voltage" of base-emitter, injection of electrons generated through strain/stress from emitter to the base occurs since the base-emitter junction will be on. Injected electrons will be swept to collector due to extension of collector-base junction capacitance towards the base. This transient bipolar action results in additional conduction current ($I_{CE\ Conduction}(t)$). Therefore, amplification of signal is justified since $I_{CE}(t) = I_{CE\ Dis}(t) + I_{CE\ Cond}(t)$.

To verify the charge injection from the emitter to the base, potential across the base-emitter has been recorded under applied stress. It is perceived that the generated charges at emitter side injects into the base and consequently a potential is observed across the base-emitter. Moreover, base-emitter voltage has a relation with the applied pressure. As it can be seen from Figure 1, increment in applied pressure results in enhancement of base-emitter voltage which is due to injection of piezoelectric charge generation from emitter side. PBJT differs from traditional BJT in transient response in that "control" over the base is occurred through applied strain/stress and generated charges sequentially.

As it can be seen from Figure S4-2, increment in applied pressure results in enhancement of base-emitter voltage which is due to injection of piezoelectric charge generation from emitter side to

the base. PBJT differs from traditional BJT in transient response in that “control” over the base is occurred through applied pressure and generated charges sequentially.

When $V_{BE}<V_{BE\ ON}$ “turn-on voltage”, base-emitter junction is off and the electron injection from emitter to base is weak.

When $V_{BE}=V_{BE\ ON}$ “turn-on voltage” of base-emitter, the emitter-base junction will be ON and the generated charges from emitter will be injected to the base. Thereafter, due to extension of collector-base junction capacitance to the base effectively, injected charges will be swept to the collector. This bipolar action adds up conduction current to $I_{CE}(t)$ additional to displacement current as discussed earlier. In this situation, it is assumed that device works in saturation region.

When $V_{BE}>>V_{BE\ ON}$, Injected charges to the base region will increase drastically which results in better signal amplification. In this scenario, device works in active region, output voltage will be enhanced due to piezo-charge generation which is dominant where a low-pressure is applied ($\frac{dV_{CE}(t)}{dt} = \frac{d(\frac{Q}{C})(t)}{dt}$).

Supplementary Information S5: Simulation of PBJT as a sensory unit

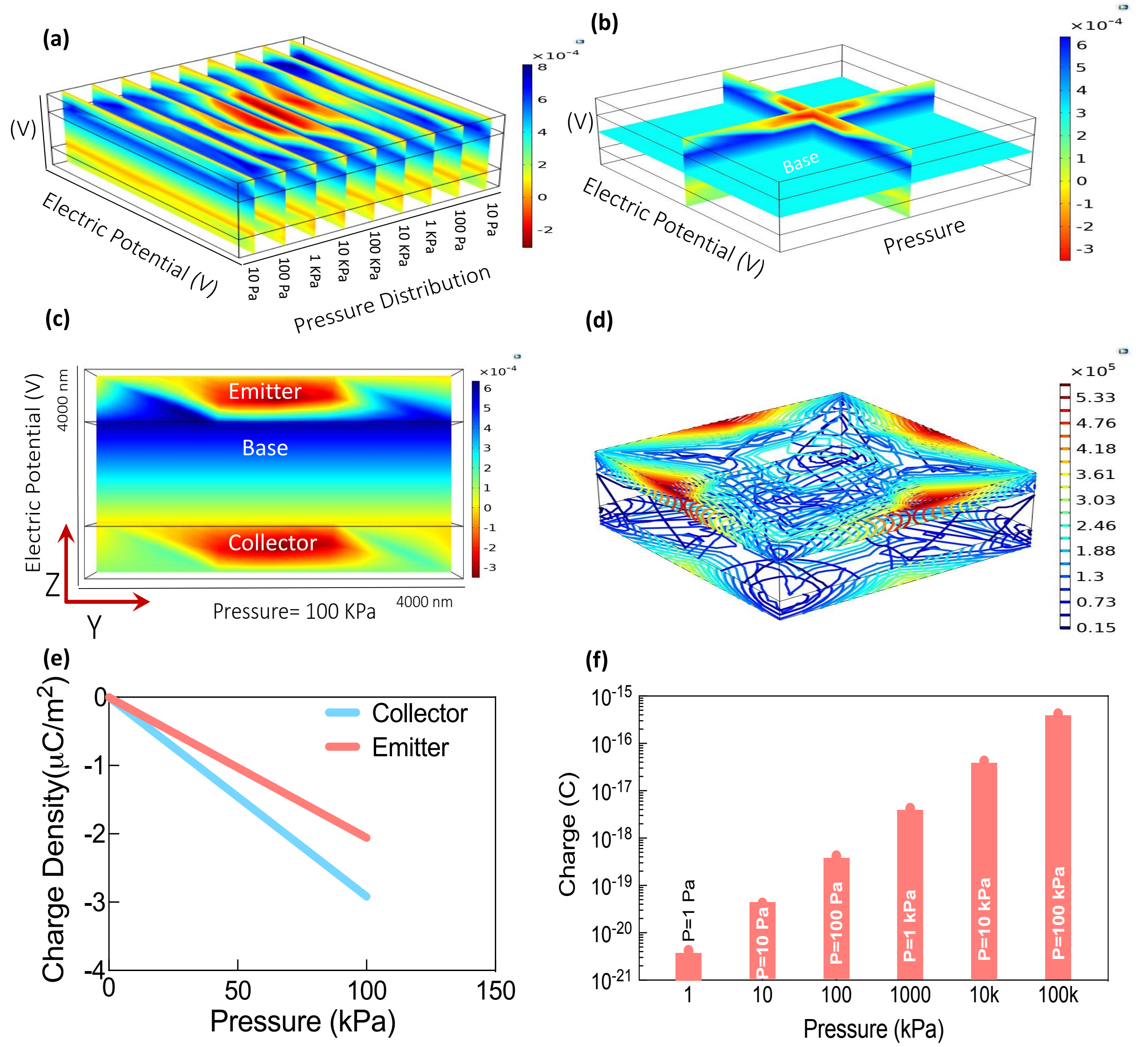

Figure S5. Simulation modeling of piezotronic n-p-n bipolar transistor as a sensory unit. Photograph of in-plane 3D piezoelectric potential generated due to applied pressure **a)** X-Y-Z plane, **b)** Intersection of X-Y planes. **c)** Electric potential of a unique plane of Z-Y, **d)** Von Mises stress distribution under 100 kPa applied pressure. **e)** Generated charge density versus the applied pressure for collector and emitter. **f)** Increment trend of total accumulated induced charges of devices as the pressure increases.

To investigate the behavior of pieziotronic bipolar transistor in static and dynamic mode, a simulation has been given and the correspondent charge generation, deformation, stress distribution and in-plane piezoelectric potential have been analyzed through COMSOL

Multiphysics. A detail study of PBJT from sensing stand point with respect to simulation is given in this section. Figure S5a schemes distribution of pressure and electric potential in X-Y-Z planes assuming a p type polymer (base) which is sandwiched by two ZnO layers classifying as ZnO (emitter) and ZnO (collector). Figure S5b illustrates the distribution of generated electric potential out of a particular direction where two planes intersect as the applied pressure is exerted atop. Figure S5c depicts the induced electric potential distribution correspondent to the applied pressure (100 kPa) in a Y-Z plane. Von mises stress distribution is illustrated in Fig. S5d correspondent to the applied pressure of 100 kPa. Charge density ($\mu C/m^2$), of each piezotronic layer as the pressure stimuli is applied are recognized by Fig. S5e. Accumulation of total induced piezoelectric charges in (C) with respect to applied pressure is shown in Fig. S5f.

Supplementary Information S6: Details of experimental data extracted by PBJT as a pressure sensor

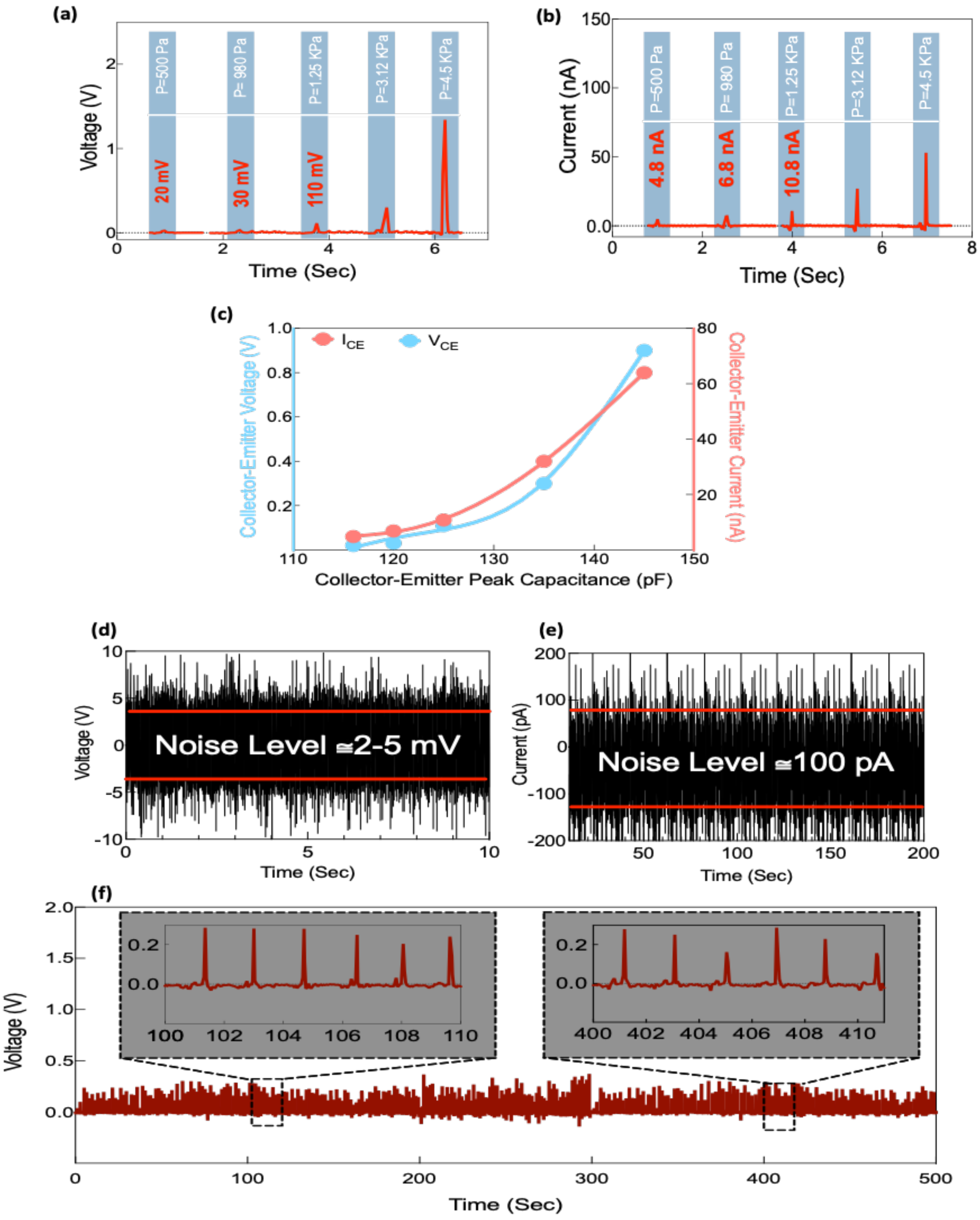

Fig. S6. Piezotronic n-p-n bipolar junction transistor dynamic behavior regarding to voltage, current and capacitance. a) Transient voltage response of collector-emitter under various applied pressures, **b)** Transient current response of collector-emitter under various applied pressures, **c)** Dynamic collector-emitter voltage/current versus capacitance of collector-emitter. Extraction of

noise level for **d)** Voltage **e)** Current. Dynamic current versus capacitance of collector-emitter variation under applied pressure, **f)** Stability of device under 300 cycles of working (frequency is almost 5 Hz and applied pressure is in range of 500 Pa.

Piezotronic n-p-n transistor is investigated under dynamic stimuli with respect to current, voltage, and capacitance. Also, stability of device is verified through 300 cycles of working under frequency of 5 Hz and almost 500 Pa (pressure). Figure S6a-b showcases the transient response of piezotronic n-p-n bipolar junction transistor based on voltage and current where various pressure is applied towards the emitter respectively. Increasing trend of collector-emitter peak capacitance with respect to transient voltage/current is illustrated in Fig. S6c. To investigate the noise level as the device is connected to oscilloscope/multimeter Fig. S6d-e are given respectively. Stability of proposed sensory unit always matters in niche applications. Therefore, the device under low frequency of almost 5 Hz and pressure of almost 500 Pa (through rotating shaft-like device) is depicted in Fig. S6f.

Supplementary Information S7: Details of experimental data extracted by a p-n junction diode as a pressure sensor

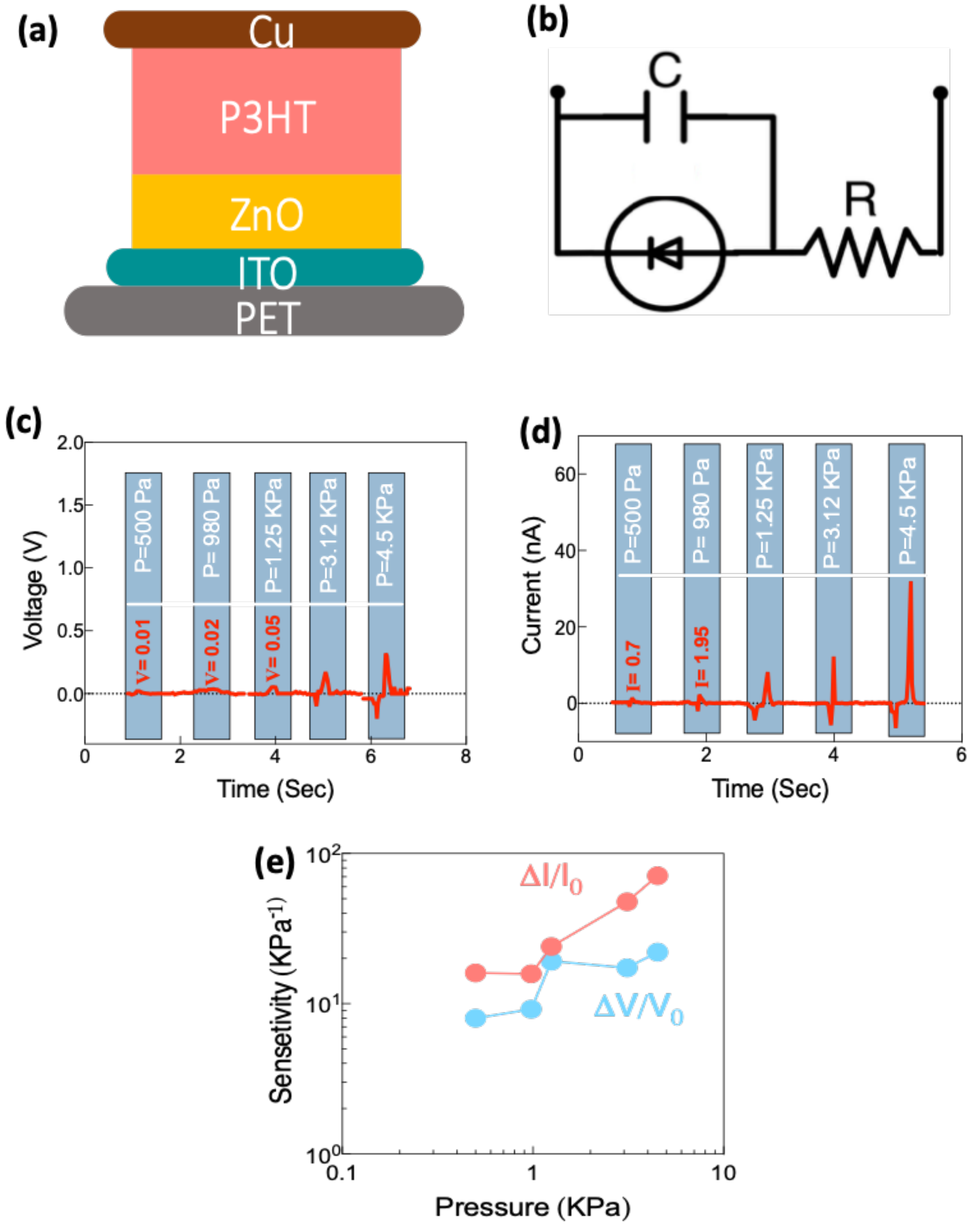

Fig. S7. n-p diode as a sensory unit. a) Schematic of n-p junction diode, **b)** Electrical equivalent circuit of n-p diode, **c)** Extracted voltage of device under various applied pressure, **d)** Current extraction of device analogous to part b. **e)** Sensitivity of n-p diode.

Fabricated n-p diode device is analyzed and the responses of device is given as a pressure sensory unit. Figure S7a shows the schematic of n-p diode while the electrical circuit icon is given in Fig. S7b. Voltage and current transient response of the device are both given in Fig. S7c and 6d correspondent to various applied pressures. Sensitivity of n-p junction diode is extracted (based on voltage and current) and depicted in Fig. S7e. The sensitivity of device is obtained through given data in Fig. 4c and d in the main manuscript ($\Delta I/I_0$, and $\Delta V/V_0$ versus pressure).

References

[1] Jäckle, S. et al. Junction formation and current transport mechanisms in hybrid n-Si/PEDOT:PSS solar cells. *Sci. Rep.* **5**, 13008 (2015).

[2] He, Z. et al. Conjugated Polymer Controlled Morphology and Charge Transport of Small-Molecule Organic Semiconductors. *Sci. Rep.* **10**, 4344 (2020).

[3] Sun, K. et al. Review on application of PEDOTs and PEDOT:PSS in energy conversion and storage devices. *J. Mater. Sci.: Mater. Electron.* **26**, 4438–4462 (2015).

[4] Xu, L. et al. Conjugated molecule doping of triphenylamine-based hole-transport layer for high-performance perovskite solar cells. *Journal of Power Sources* **506**, 230120, (2021).

[5] Pallavi, Sh. et al. Development and characterization of confocal sputtered piezoelectric zinc oxide thin film. *Vacuum* **184**, 109930, (2021).

[6] Polewczyk, V. et al. ZnO Thin Films Growth Optimization for Piezoelectric Application. *Sensors* **21**, 6114 (2021).

[7] Molarius, J. et al. Piezoelectric ZnO films by r.f. sputtering. *Journal of Materials Science: Materials in Electronics* **14**, 431–435 (2003).

[8] Gardeniers, J. et al. Preferred orientation and piezoelectricity in sputtered ZnO films. *Journal of Applied Physics* **83**, 7844–7854 (1998).

[9] Wen, X. et al. Development and progress in piezotronics. *Nano Energy* **14**, 276-295 (2015).

[10] K.Y. Lee, et al. p-Type polymer-hybridized high-performance piezoelectric nanogenerators. *Nano Lett*. **12**, 4 (2012).

[11] M. Sajedi Alvar, et al. Space-charge-limited electron and hole currents in hybrid organic-inorganic perovskites. *Nat. Commun.* **11**, 4023 (2020).
